# Validation of the STOP-Bang Questionnaire as a Screening Tool for Obstructive Sleep Apnea among Different Populations: A Systematic Review and Meta-Analysis

**DOI:** 10.1371/journal.pone.0143697

**Published:** 2015-12-14

**Authors:** Mahesh Nagappa, Pu Liao, Jean Wong, Dennis Auckley, Satya Krishna Ramachandran, Stavros Memtsoudis, Babak Mokhlesi, Frances Chung

**Affiliations:** 1 Department of Anesthesiology, Toronto Western Hospital, University Health Network, University of Toronto, Toronto, Ontario, Canada; 2 Division of Pulmonary, Critical Care and Sleep Medicine, Metro Health Medical Center, Case Western Reserve University, Cleveland, OH, United States of America; 3 Department of Anesthesiology, University of Michigan Health System, East Medical Center Drive, Michigan, United States of America; 4 Department of Anesthesiology, Hospital for Special Surgery, Weill Medical College of Cornell University, New York, United States of America; 5 Department of Medicine, Sleep Disorders Center and the Section of Pulmonary and Critical Care, University of Chicago, Chicago, Illinois, United States of America; Hospital General Dr. Manuel Gea González, MEXICO

## Abstract

**Background:**

Diagnosing obstructive sleep apnea (OSA) is clinically relevant because untreated OSA has been associated with increased morbidity and mortality. The STOP-Bang questionnaire is a validated screening tool for OSA. We conducted a systematic review and meta-analysis to determine the effectiveness of STOP-Bang for screening patients suspected of having OSA and to predict its accuracy in determining the severity of OSA in the different populations.

**Methods:**

A search of the literature databases was performed. Inclusion criteria were: 1) Studies that used STOP-Bang questionnaire as a screening tool for OSA in adult subjects (>18 years); 2) The accuracy of the STOP-Bang questionnaire was validated by polysomnography—the gold standard for diagnosing OSA; 3) OSA was clearly defined as apnea/hypopnea index (AHI) or respiratory disturbance index (RDI) ≥ 5; 4) Publications in the English language. The quality of the studies were explicitly described and coded according to the Cochrane Methods group on the screening and diagnostic tests.

**Results:**

Seventeen studies including 9,206 patients met criteria for the systematic review. In the sleep clinic population, the sensitivity was 90%, 94% and 96% to detect any OSA (AHI ≥ 5), moderate-to-severe OSA (AHI ≥15), and severe OSA (AHI ≥30) respectively. The corresponding NPV was 46%, 75% and 90%. A similar trend was found in the surgical population. In the sleep clinic population, the probability of severe OSA with a STOP-Bang score of 3 was 25%. With a stepwise increase of the STOP-Bang score to 4, 5, 6 and 7/8, the probability rose proportionally to 35%, 45%, 55% and 75%, respectively. In the surgical population, the probability of severe OSA with a STOP-Bang score of 3 was 15%. With a stepwise increase of the STOP-Bang score to 4, 5, 6 and 7/8, the probability increased to 25%, 35%, 45% and 65%, respectively.

**Conclusion:**

This meta-analysis confirms the high performance of the STOP-Bang questionnaire in the sleep clinic and surgical population for screening of OSA. The higher the STOP-Bang score, the greater is the probability of moderate-to-severe OSA.

## Introduction

Obstructive sleep apnea (OSA) is a prevalent sleep breathing disorder affecting 9–25% of the general adult population.[1] It is associated with cardiovascular diseases, cerebrovascular diseases, metabolic disorders and impaired neurocognitive function.[2–4] It has been estimated that up to 80% of individuals with moderate-to-severe OSA may remain undiagnosed.[5] The prevalence is higher in the surgical population,[6,7] with a prevalence rates as high as 70% in bariatric surgical patients.[8,9] The majority of surgical patients with OSA remain undiagnosed and subsequently, are untreated at the time of presentation for surgery.[7] Given the important adverse consequences associated with untreated OSA, prompt diagnosis and treatment of unrecognized OSA is critical. The gold standard for diagnosis of OSA is an overnight polysomnogram (PSG). However, PSG is time consuming, labor intensive, and costly. Moreover, PSG requires the expertise of sleep medicine specialists, which may not be readily available at many hospitals and medical centers. Therefore, a simple and reliable method of identifying patients who are at high-risk of OSA and triaging them for prompt diagnosis and treatment is clinically relevant. A number of screening tests have been developed to identify high-risk patients.[10–16] However, many of these screening tests are lengthy and complicated, or require an upper airway assessment, making them inconvenient to use and may increase variability amongst clinicians performing the upper airway assessment.

The STOP-Bang questionnaire was first developed in 2008.[17] It is a simple, easy to remember, and self-reportable screening tool, which includes four subjective (STOP: **S**noring, **T**iredness, **O**bserved apnea and high blood **P**ressure) and four demographics items (Bang: **B**MI, **a**ge, **n**eck circumference, **g**ender).[17] The STOP-Bang questionnaire was originally validated to screen for OSA in the surgical population. The sensitivity for the STOP-Bang score ≥3 as the cut-off to predict any OSA (apnea hypopnea index (AHI) >5), moderate-to-severe OSA (AHI >15) and severe OSA (AHI >30) was 83.9%, 92.9% and 100% respectively.[17] Due to its ease of use and high sensitivity, the STOP-Bang questionnaire has been widely used in preoperative clinics[17–19], sleep clinics[20–30], the general population[31] and other special populations[32,33] to detect patients at high-risk of OSA. The purpose of this systematic review and meta-analysis is to determine the accuracy of the STOP-Bang questionnaire in screening patients for OSA and to evaluate the relationship between the STOP-Bang score and the probability of OSA among different patient populations.

## Methods

### Literature search strategy and study selection

We identified and reviewed published articles in which the STOP-Bang questionnaire was assessed as a screening tool for OSA among different patient populations. The literature search was performed according to PRISMA (Preferred Reporting Items for Systematic Reviews and Meta-analysis) guidelines and the search strategy was implemented with the help of an expert librarian familiar with the literature search.

#### Electronic searches

All queries started in 2008 when the STOP-Bang was first published.[17] With the goal of completeness, a systematic search of the literature was carried out using multiple sources, including MEDLINE (from 2008 to January 2015), Medline-in-process & other non-indexed citations (up to January 2015), Embase (from 2008 to January 2015), Cochrane Central Register of Controlled Trials (up to January 2015), Cochrane Databases of Systematic Reviews (from 2008 to January 2015), Google Scholar, Web of Sciences (from 2008 to January 2015), Scopus (from 2008 to January 2015) and PubMed (from 2008 to January 2015) using the search strategy that was designed for each database. The search strategy included the following free-text and index terms: ‘obstructive sleep apnea’, ‘obstructive sleep apnea syndrome’, ‘obstructive sleep apnoea’, ‘obstructive sleep apnoea syndrome’, ‘sleep disordered breathing’, ‘obesity hypoventilation syndrome’, ‘apnea or apnoea’, ‘hypopnea or hypopnoea’, ‘STOP-Bang’, ‘STOP Questionnaire’.

#### Searching other resources

A citation search was also conducted by performing a manual review of references from the final articles analyzed as well as the related review articles.

#### Selection of studies

Two reviewers (M.N., F.C.) independently screened the titles and abstracts of the search results. After excluding the irrelevant articles, full-text articles of the remaining publications were retrieved and carefully evaluated to determine if they met the following inclusion criteria: 1) The study evaluated the STOP-Bang questionnaire as a screening tool for OSA in adult subjects >18 years; 2) The results of a PSG (either laboratory or portable) confirming the diagnosis of OSA; 3) OSA and its severity was defined by an AHI or a respiratory disturbance index (RDI); and 4) The full-text papers were written in the English language.

### Data extraction and management

The two independent reviewers (M.N. & F.C.) extracted the data with a standard data collection form. For each study, a 2X2 contingency table was constructed using the predictive parameters for each AHI or RDI cut-off. Studies were excluded if there was inadequate information to create the 2x2 contingency tables or if there was an inadequate description of the methodology. The duplicates were removed and any disagreements were resolved by consulting with another author (P.L.). Unless specifically defined, the standard cut-off of the STOP-Bang questionnaire (STOP-Bang ≥3) was adopted. An AHI ≥5 or RDI ≥5 were considered as the diagnostic cut-off for OSA. An AHI ≥15 or RDI ≥15 were considered as the diagnostic cut-off for moderate-to-severe OSA, and AHI ≥30 or RDI ≥30 for severe OSA.

The following information was collected from each study: author, year of publication, type of study, type of patients (surgical patients, sleep clinic patients, general population, renal failure patients and highway bus drivers), sample size, validation process and tool, OSA definition and number of patients in each of the following categories: mild (AHI ≥5), moderate-to-severe (AHI ≥15 or RDI ≥15) and severe OSA (AHI ≥30 or RDI ≥30). The following clinic data were also extracted: age, gender, Body Mass Index (BMI), neck circumference, the STOP-Bang score, mean AHI/RDI and minimum SpO_2_.

### Assessment of methodological quality

The methodological quality of each study was assessed and any disagreements were resolved by consulting another author (PL). The validity criteria assessing the internal and external validity were explicitly described and coded according to the Cochrane Methods group on the screening and diagnostic tests.[34] The internal validity included the following factors: study design, definition of the disease, blind execution of the index test (STOP-Bang questionnaire) and the reference test (PSG), valid reference test, avoidance of verification bias, and independent interpretation of the test results. The external validity consisted of the following items: disease spectrum, clinical setting, demographic information, previous screening or referral filter, explicit cutoffs, percentage of missing patients, missing data management, and subject selection for PSG.

### Statistical analysis

The continuous data are presented as mean and standard deviation and categorical data as frequency and percentage. Using 2X2 contingency tables, we recalculated the following predictive parameters in each study: prevalence, sensitivity and specificity, positive predictive value (PPV) and negative predictive value (NPV), and diagnostic odds ratio (DOR). The area under the receiver operating characteristic (ROC) curve were calculated by logistic regression. The pooled predictive parameters (sensitivity and specificity, positive and negative predictive value, DOR and area under the ROC curve were obtained to assess the performance of each STOP-Bang score for the different AHI cut-offs (AHI ≥5, AHI ≥15 and AHI ≥30). The probability of moderate and severe OSA at the various STOP-Bang scores were pooled and presented as a bar graph.

The meta-analysis was carried out with Review Manager Version 5.3. Copenhagen (The Nordic Cochrane Centre, The Cochrane Collaboration, 2014) and Meta-Disc version 1.4 (Hospital Ramony Cajal, Madrid, Spain). The parameters were assessed separately for each population with similar characteristics (i.e. sleep clinic population, surgical population). The parameters from pooled data of each population were calculated and forest plots were created for the predictive parameters using a random effect model. DOR and ROC curve analysis was presented to assess the diagnostic ability of STOP-Bang questionnaire. Inconsistency was assessed using the Cochrane Q test (P value <0.05: heterogeneity present) and I^2^ test (I^2^ >33%: heterogeneity present).

## Results

**Fig 1:**
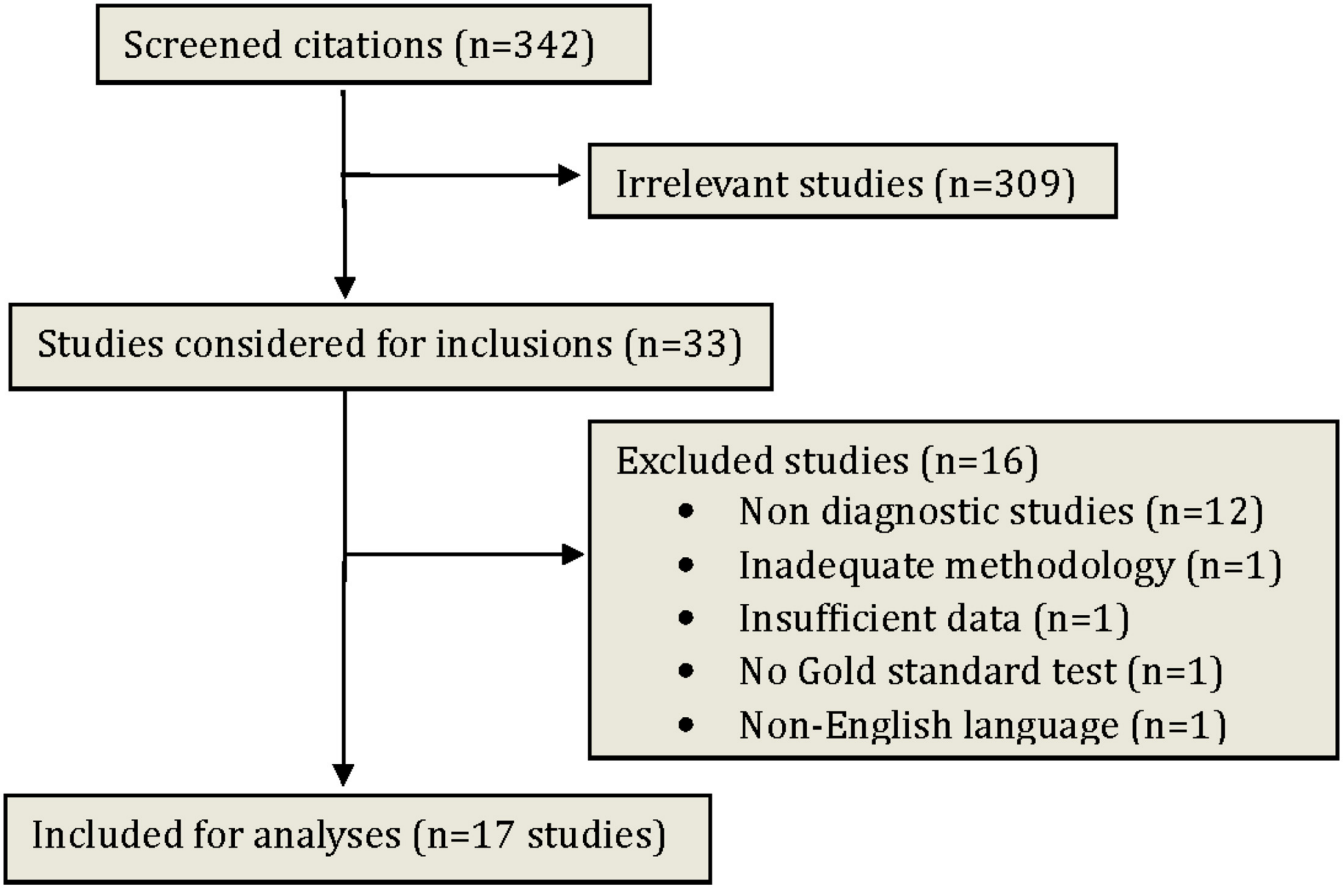
Flow chart of screened, excluded and analyzed studies.

Our initial search yielded 342 citations (Fig 1). After screening titles and abstracts, 309 studies were excluded due to not meeting the predetermined eligibility criteria. Of the remaining 33 studies, 16 studies were excluded and the reasons are listed in S1 Appendix.[35–50] Finally, seventeen studies were included in the review.[17–33] The included studies encompassed 9,206 patients and were conducted in nine different countries: Canada[17,18,25,33], USA[21,24,26,31], China[23,28,29] Brazil[19], Egypt[22], Singapore[20], Turkey[32], Portugal[30] and United Kingdom[27].

**Fig 2:**
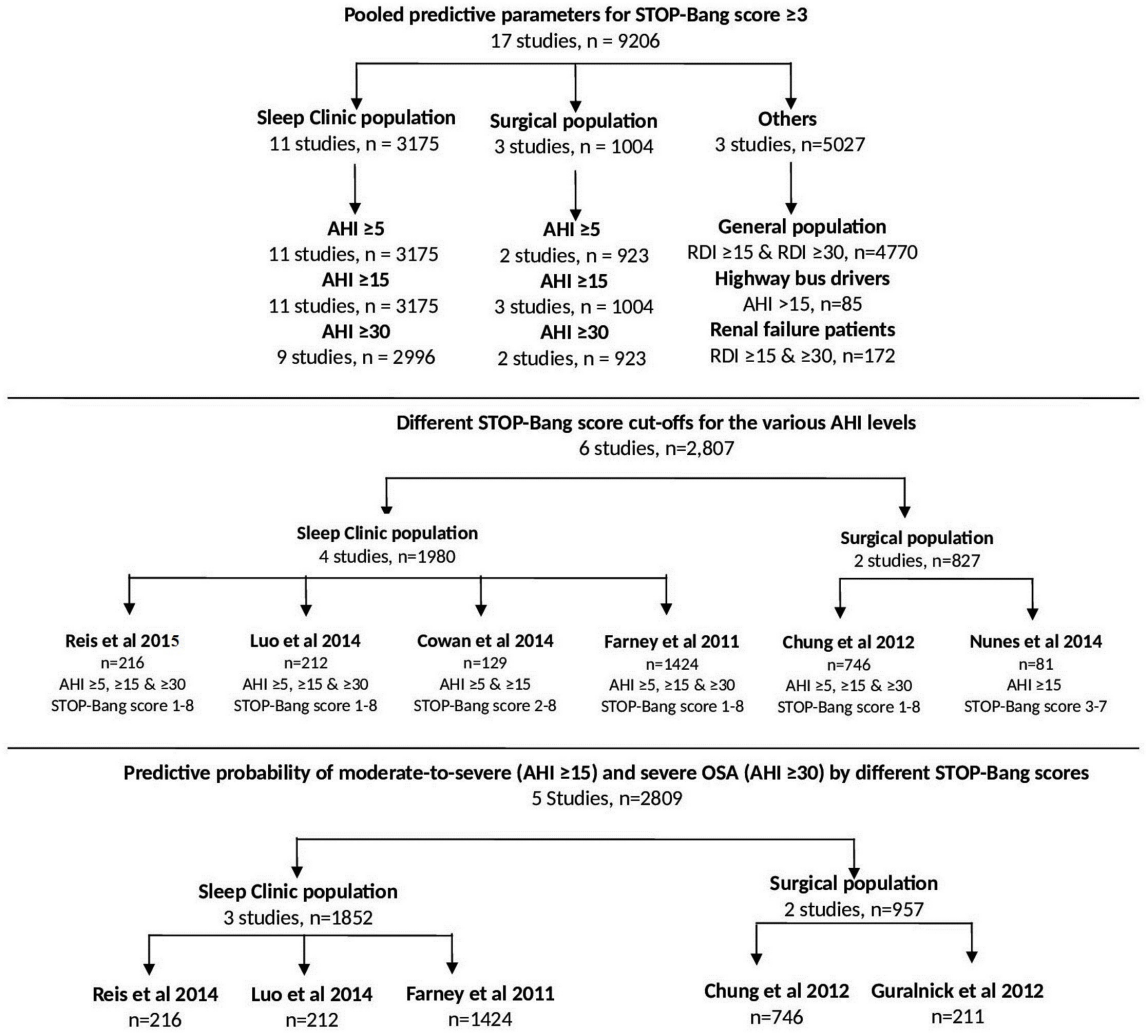
Flow chart for data collection and grouping of the studies for systematic review and meta-analysis. AHI: Apnea-Hypopnea Index; RDI: Respiratory Disturbance Index; n: number of participants.

These 17 studies were included for systematic review. Out of which 11 studies in Sleep clinic population and 3 studies in surgical population are included for meta-analysis. Among the sleep clinic population, 11 studies (n = 3176)[20–30] were included for meta-analysis at the AHI cut-offs of ≥5 and ≥15, and 9 studies (n = 2996)[20–25,28–30] for AHI ≥30. Among the surgical population, two studies (n = 923)[17,18] were included for AHI ≥5, three (n = 1004)[17–19] for AHI ≥15, and two (n = 923)[17,18] for AHI ≥30 (Fig 2). The information regarding the number of patients and the AHI validation among the general population[31], highway bus drivers[32] and renal failure patients[33] are also listed in Fig 2. For the meta-analysis, pooling of the data was performed within populations with similar characteristics (i.e. sleep clinic population and surgical population).

The validation characteristics and demographic data (expressed as mean±SD) of the included studies are summarized in Tables 1 and 2 respectively. The majority were prospective studies, in which patients completed the STOP-Bang questionnaire before undergoing PSG with only two being retrospective studies[21,31]. There were variations in the cut-off criteria for defining OSA with fourteen studies using AHI ≥5 [17,18,20–30]. Two studies each defined OSA as either AHI ≥15[19,32], or RDI ≥15 respectively.[31,33].

**Table 1:** The characteristics of the included studies.

Study	Type (n)	Validation process & Tool	Prevalence	OSA definition	No OSA AHI <5 (n)	Mild OSA AHI ≥5 (n)	Moderate-to-severe OSA AHI ≥15 or RDI >15 (n)	Severe OSA AHI ≥30 or RDI ≥30 (n)
**Sleep clinic population**								
^[^ ^20^ ^]^Ong ^2010^	Asian population (319)	Prospective Lab PSG	77.7	AHI >5	79	240	166	113
^[^ ^21^ ^]^Farney^2011^	American population (1426)	Retrospective Lab PSG	89.5	AHI ≥5	150	1274	959	580
^[^ ^22^ ^]^El-Sayed^2012^	Egyptian population (234)	Prospective Lab PSG	87.2	AHI ≥5	30	204	177	148
^[^ ^23^ ^]^Yu^2012^	Chinese population (114)	Prospective Lab PSG	79.8	AHI ≥5	23	91	67	46
^[^ ^24^ ^]^Boynton^2013^	American population (219)	Prospective Lab PSG	77.1	AHI >5	50	169	103	62
^[^ ^25^ ^]^Pereire^2013^	Canadian population (128)	Prospective Lab PSG	91.3	AHI >5	12	116	88	56
^[^ ^26^ ^]^Vana^2013^	American population (47)	Prospective Lab PSG	68.0	AHI >5	15	32	19	9
^[^ ^27^ ^]^Cowan^2014^	British population (129)	Prospective Portable level 3 PSG	75.2	AHI ≥5	32	97	56	NA
^[^ ^28^ ^]^Ha^2014^	Chinese population (139)	Prospective Lab PSG	79.8	AHI >5	28	111	84	51
^[^ ^29^ ^]^Luo^2014^	Chinese population (194)	Prospective Lab PSG	92.4	AHI ≥5	8	186	170	128
^[^ ^30^ ^]^Reis^2015^	Portuguese population (216)	Prospective Lab/portable level 3 PSG	78.1	AHI >5	47	168	113	61
**Surgical population**								
^[^ ^17^ ^]^Chung^2008^	Canadian population (177)	Prospective Lab PSG	68.9	AHI >5	55	122	70	39
^[^ ^18^ ^]^Chung^2012^	Canadian population (746)	Prospective Lab/portable level 2 PSG	68.4	AHI >5	236	510	287	134
^[^ ^19^ ^]^Nunes^2014^	Brazilian population (81)	Prospective Lab PSG	48.1	AHI ≥15		AHI <15 = 43	38	NA
**General population**								
^[^ ^31^ ^]^Silva^2011^	American population (4770)	Retrospective Portable level 2 PSG	12.7	RDI ≥15		RDI<15 = 3822	948	345
**Highway bus drivers**								
^[^ ^32^ ^]^Firat^2012^	Turkish population (85)	Prospective Lab PSG	54.1	AHI >15		AHI<15 = 39	46	NA
**Renal failure patients**								
^[^ ^33^ ^]^Nicholl^2013^	Canadian population (172)	Prospective Portable level 3 PSG	42.4	RDI ≥15		AHI<15 = 99	73	50

**Table 2:** Demographic data of patients using STOP-Bang questionnaire.

Study ID	No. of patients	Age (Year)	Gender (%) Male/Female	BMI (Kg/m^2^)	Neck Cir (cm)	STOP-Bang Score	AHI (mean)	Minimum SPO2 (%)
**Sleep Clinic population**								
^[^ ^20^ ^]^Ong^2010^	314	46.8±15	70/30	27.9±6	39.8±4	3.8±2	26.2±27	82±13
^[^ ^21^ ^]^Farney^2011^	1426	49.7±15	57/43	33.8±8	40.7±5	4.3±2	32.9±30	NA
^[^ ^22^ ^]^El-Sayed^2012^	234	50.3±11	85/15	37.7±1	42.4±4	5.6±2	45.6±33	NA
^[^ ^23^ ^]^Yu^2012^	114	40.5±02	89/11	28.2±2	39.5±3	3.8±1	NA	NA
^[^ ^24^ ^]^Boynton^2013^	219	46.3±14	44/56	33.4±9	39.9±5	3.9±2	NA	NA
^[^ ^25^ ^]^Pereire^2013^	128	50.0±12	65/35	31.0±7	41.0±4	NA	33.1±28	NA
^[^ ^26^ ^]^Vana^2013^	47	46.4±13	34/66	36.3±9	38.1±5	5.0±2	08.9±24	NA
^[^ ^27^ ^]^Cowan^2014^	129	49.0±11	63/37	32.0±6	NA	NA	NA	NA
^[^ ^28^ ^]^Ha ^2014^	139	45.0±11	82/18	26.0±4	NA	NA	25.0±24	80±9
^[^ ^29^ ^]^Luo ^2014^	212	44.8±12	88/12	28.1±4	41.1±3	4.4±1	43.7±02	74±13
^[^ ^30^ ^]^Reis^2015^	215	53.6±13	71/29	29.0±5	40.4±44	4.4±2	16.7±20	NA
**Surgical population**								
^[^ ^17^ ^]^Chung^2008^	177	56.0±13	60/40	30.0±6	39±6	NA	20.0±06	82±11
^[^ ^18^ ^]^Chung^2012^	746	60.0±11	49/51	30.0±6	NA	NA	NA	NA
^[^ ^19^ ^]^Nunes^2014^	81	56.0±07	70/30	29.5±5	NA	NA	NA	NA
**General population**								
^[^ ^31^ ^]^Silva^2011^	4770	62.4±10	51/49	NA	NA	3.4±1	NA	NA
**Highway bus drivers**								
^[^ ^32^ ^]^Firat^2012^	85	NA	NA	29.1±4	41.1±3	NA	21.1±17	NA
**Renal failure patients**								
^[^ ^33^ ^]^Nicholl^2013^	172	63.0±13	63/37	29.3±7	41±5	4±1.5	RDI = 13.5	NA

### Methodological quality of the included studies

All included studies used PSG as a valid reference test to verify the accuracy of the STOP-Bang questionnaire, confirming internal validity (S2 Appendix). For validation purposes, 12 studies used laboratory PSG[17,19–26,28,29,32], while three used portable PSG (level 3 PSG[27,33] or level 2 PSG[31] and two studies used laboratory or portable level 2/3 PSG[18,30]. All included studies had specific information to clearly evaluate the risk of bias during the validation process of the STOP-Bang questionnaire. The following aspects were available in the chosen publications: 1) Blinded interpretation of the PSG and STOP-Bang questionnaire (i.e. those who scored the PSG were unaware of the results of the STOP-Bang questionnaire and vice versa); and 2) Interpretation of the PSG results was performed independent of the patient’s clinical history. In terms of the external validity, all studies adequately met the appraisal items with one minor exception[32] (S3 Appendix). All studies clearly mentioned the inclusion and exclusion criteria.

The following abbreviations were used to evaluate the internal and external validity of the studies. F: Full meeting criteria; P: partially meeting criteria; U: Unsure if meeting criteria in subgroups; N: not meeting criteria in subgroup; N/A: not applicable.

#### Predictive parameters of STOP-Bang questionnaire in the sleep clinic population

**Fig 3:**
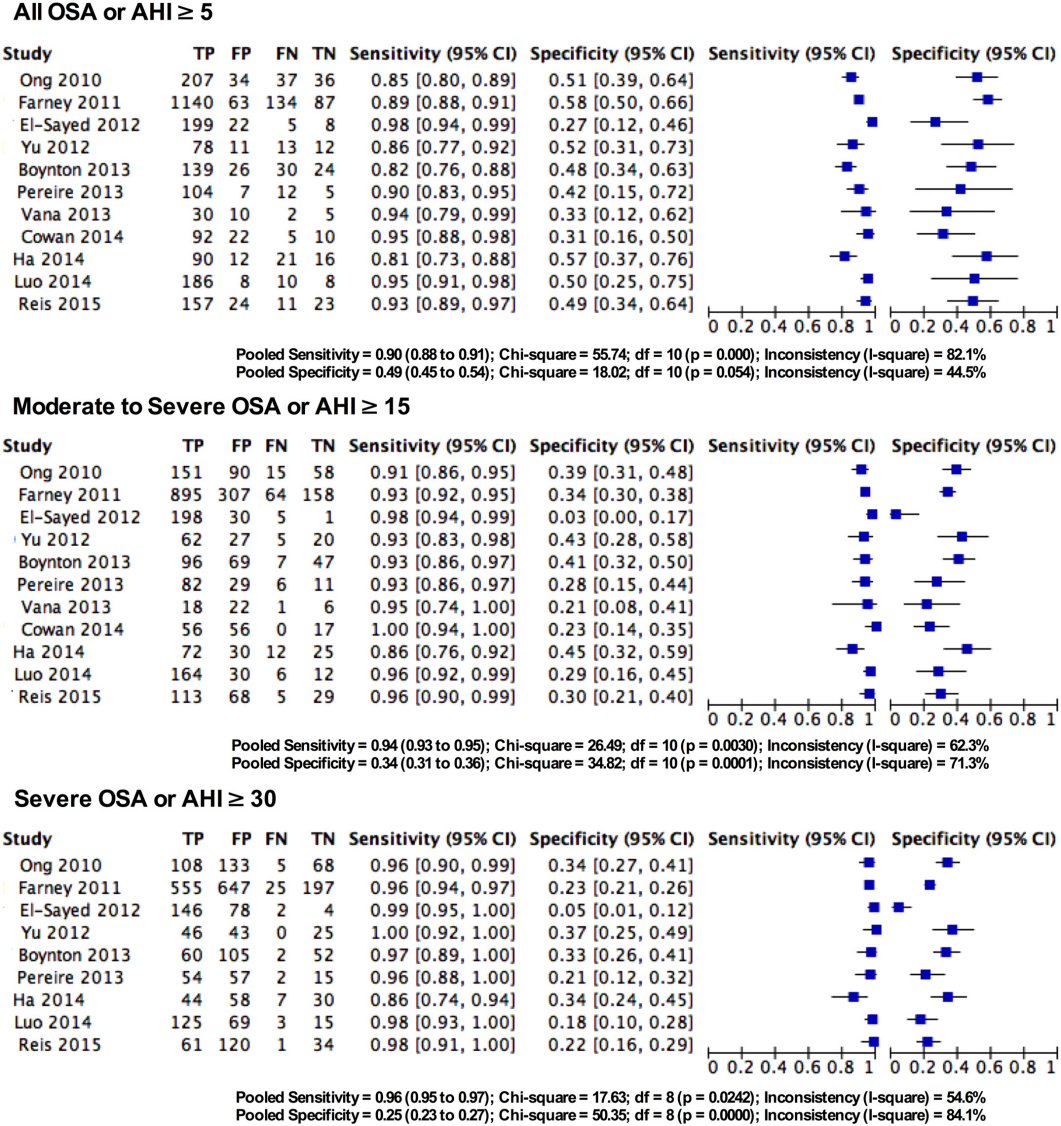
Forest plot for pooled sensitivity and specificity for various OSA severities in Sleep Clinic populations. TP—True Positive, FP—False Positive, FN—False Negative, TN—True Negative, CI—confidence Interval.

**Fig 4:**
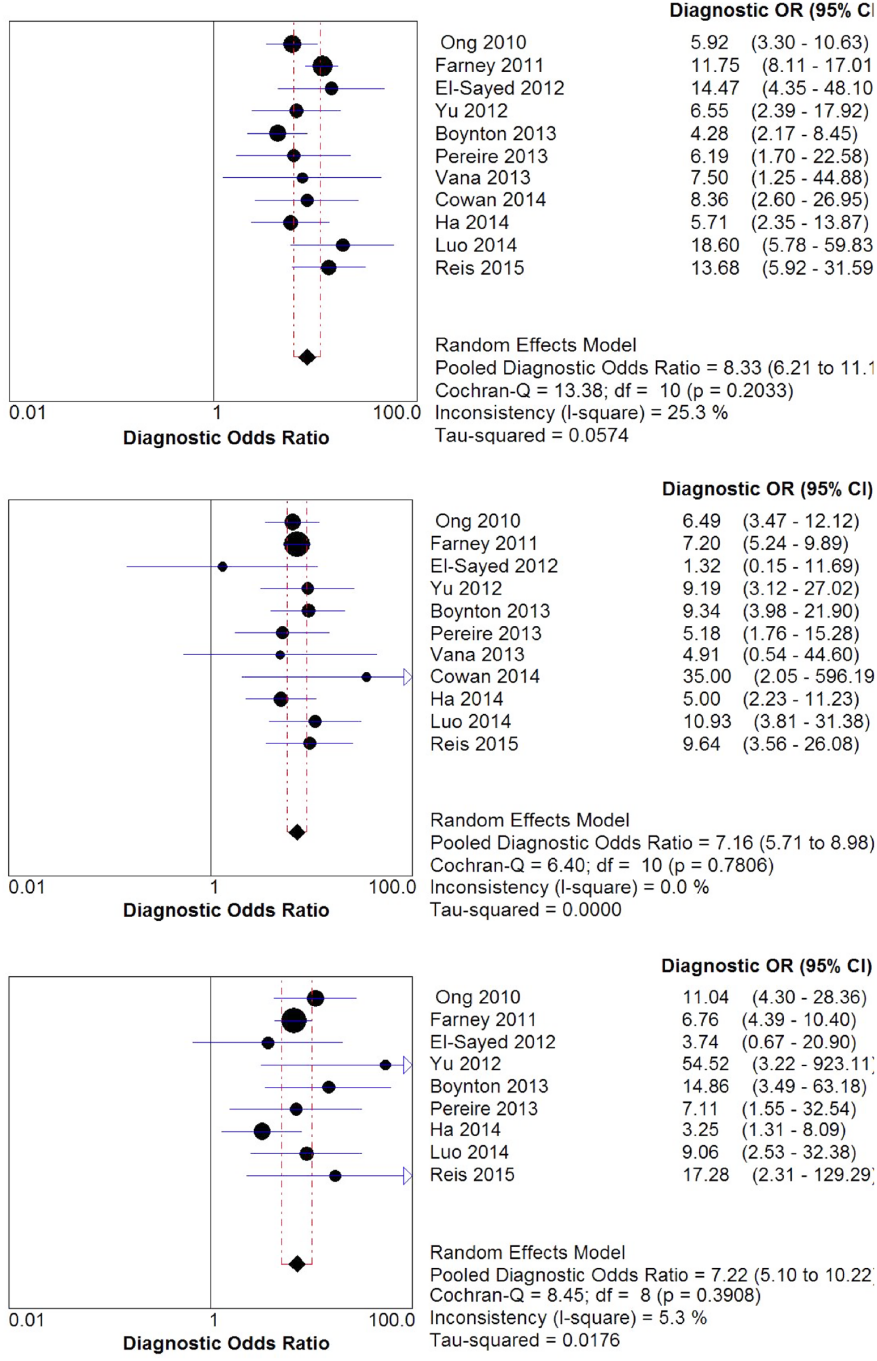
Forest plot for pooled Diagnostic odds ratio for various OSA severities in Sleep Clinic populations. OR—Odd Ratio, CI—Confidence Interval.

The pooled predictive parameters of a STOP-Bang score ≥3 as the cut-off in the sleep clinic population are presented in Table 3 and Figs 3 & 4. The prevalence of any OSA (AHI ≥5), moderate-to-severe OSA (AHI ≥15) and severe OSA (AHI ≥30) was 85%, 64% and 42% respectively. The pooled sensitivity of the STOP-Bang questionnaire to predict any OSA, moderate-to-severe and severe OSA was 90% (95%CI: 88%-91%; I^2^ = 82.9%), 94% (95%CI: 92%-95%; I^2^ = 64.8%) and 96% (95%CI: 95%-97%; I^2^ = 57.8%) respectively. The corresponding pooled NPVs were 46% (95% CI: 41%-50%), 75% (95% CI: 71%-79%) and 90% (95% CI: 87%-93%) respectively. The pooled specificity was relatively low (49%, 34% and 25% respectively). The PPV was 91%, 72% and 48% for any OSA, moderate-to-severe and severe OSA respectively. The corresponding pooled DOR were 8.3, 7.2 and 7.2 respectively. The area under the ROC curve was consistently >0.72 for all OSA severities.

**Table 3:** Pooled Predictive parameters of STOP-Bang ≥3 as cut-off.

Predictive parameters	Mild OSA AHI ≥5	Moderate-to-Severe OSA AHI ≥15	Severe OSA AHI ≥30
**Sleep clinic population**			
	(11 studies, n = 3175)	(11 studies, n = 3175)	(9 studies, n = 2996)
Prevalence	85.0 (83.0–86.0)	64.0 (62.0–65.0)	42.0 (40.0–43.0)
Sensitivity	90.0 (88.0–91.0)	94.0 (93.0–95.0)	96.0 (95.0–97.0)
Specificity	49.0 (45.0–54.0)	34.0 (31.0–36.0)	25.0 (23.0–27.0)
Positive predictive value	91.0 (90.0–92.0)	72.0 (70.0–74.0)	48.0 (46.0–50.0)
Negative predictive value	46.0 (41.0–50.0)	75.0 (71.0–79.0)	90.0 (87.0–93.0)
Diagnostic odds ratio	8.3 (6.1–9.7)	7.2 (5.7–9.0)	7.2 (5.1–10.2)
SROC	0.74	0.78	0.72
**Surgical population**			
	(2 studies, n = 923)	(3 studies, n = 1002)	(2 studies, n = 923)
Prevalence	68.0 (65.0–71.0)	39.0 (36.0–42.0)	19.0 (21.0–27.0)
Sensitivity	84.0 (81.0–87.0)	91.0 (87.0–93.0)	96.0 (92.0–98.0)
Specificity	43.0 (38.0–49.0)	32.0 (28.0–36.0)	29.0 (26.0–33.0)
Positive predictive value	76.0 (73.0–79.0)	46.0 (42.7–50.0)	23.0 (21.0–27.0)
Negative predictive value	55.0 (48.8–62.0)	84.0 (79.0–88.0)	97.0 (94.0–99.0)
Diagnostic odds ratio	4.46 (2.5–7.96)	4.08 (1.58–10.53)	11.31(2.07–61.7)
SROC	0.64	0.68	0.63

#### Predictive parameters of the STOP-Bang questionnaire in the surgical population

**Fig 5:**
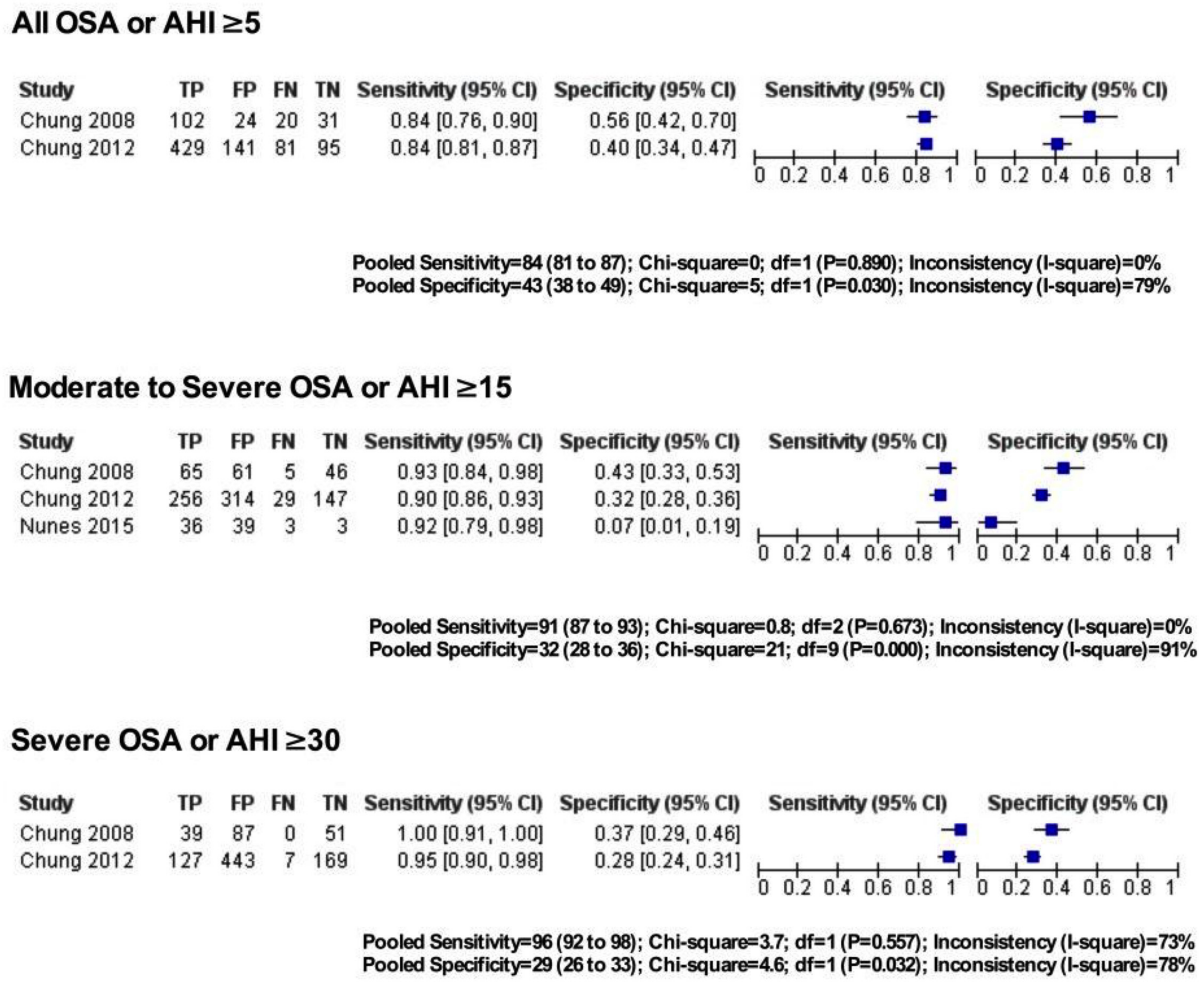
Forest plot for pooled sensitivity and specificity for various OSA severities in surgical populations. TP—True Positive, FP—False Positive, FN—False Negative, TN—True Negative, CI—confidence Interval.

**Fig 6:**
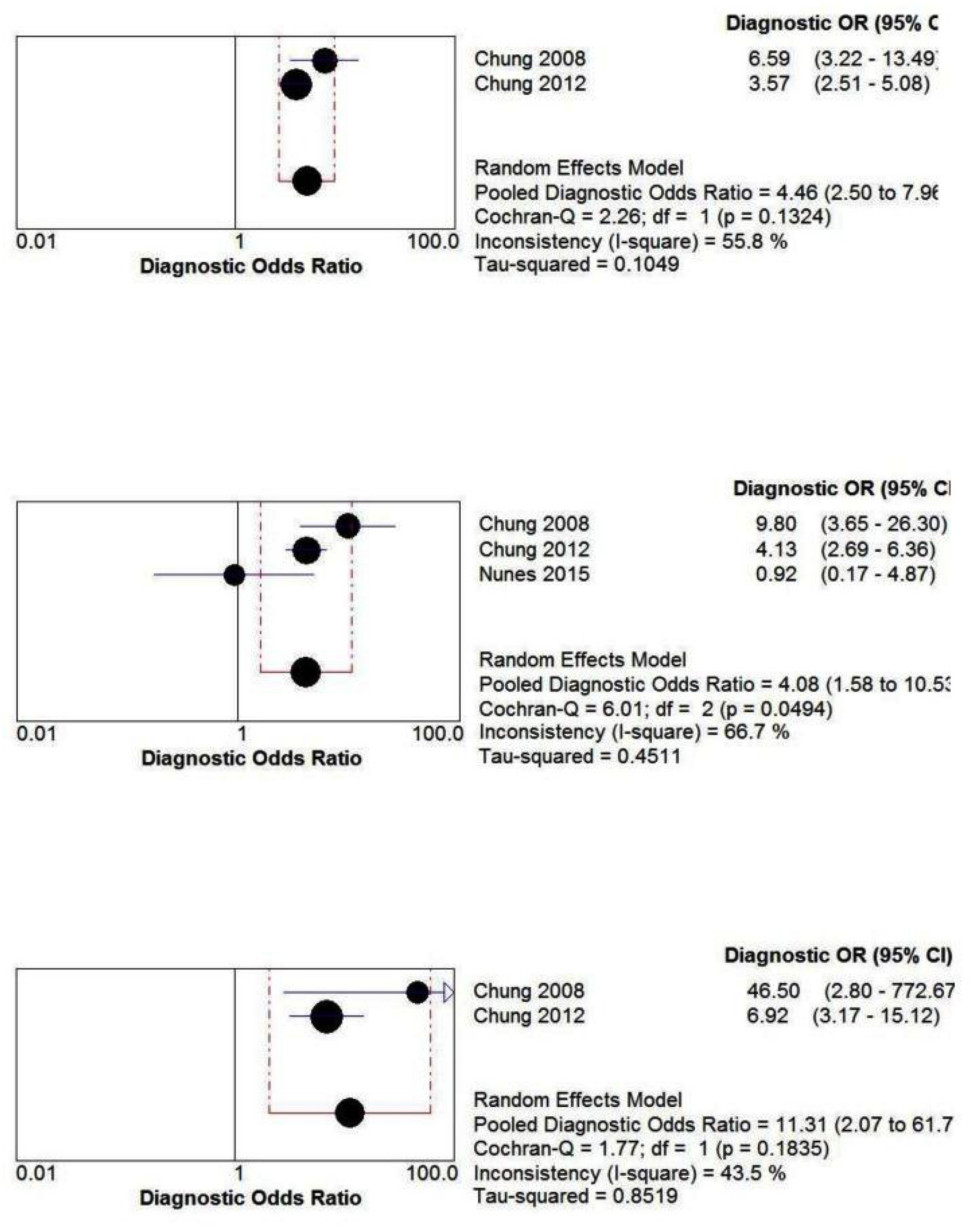
Forest plot for pooled Diagnostic Odds Ratio for various OSA severities in Surgical populations. OR—Odd Ratio, CI—Confidence Interval.

The pooled predictive parameters of a STOP-Bang score ≥3 as the cut-off in surgical patients are presented in Table 3 and Figs 5 & 6. The prevalence of OSA for any OSA, moderate-to-severe and severe OSA was 68.4%, 39.2% and 18.7% respectively. The corresponding sensitivities were 84% (95%CI: 81%-87%; I^2^ = 0%), 91% (95%CI: 87%-93%; I^2^ = 0%) and 96% (95%CI: 92%-98%; I^2^ = 72.7%) respectively; while the NPVs were 56% (95%CI: 49%-62%), 84% (95% CI: 79%-89%) and 97% (95%CI: 94%-99%) respectively. The specificity for any OSA, moderate-to-severe and severe OSA was 43%, 32% and 29% respectively, and the PPVs were 76%, 46% and 24% respectively. The corresponding DOR were 4.5, 4.0 and 11.3. The area under the ROC was consistently >0.6 for all OSA severities.

#### Predictive parameters of the STOP-Bang questionnaire in the general population

Silva et al.[31] evaluated the STOP-Bang questionnaire in 4770 participants in the Sleep Heart Health Study. The prevalence of moderate-to-severe OSA (RDI ≥15) and severe OSA (RDI ≥30) in this population was 12.7% and 7.2% respectively. The sensitivity of STOP-Bang score ≥3 as cut-off was 88% to detect moderate-to-severe OSA (RDI ≥15) and 93% to detect severe OSA (AHI ≥30). NPV was 95% and 98% respectively. The specificity remained at 30% and PPV was 16% and 9% respectively.

#### Predictive parameters of the STOP-Bang questionnaire in highway bus drivers

The STOP-Bang questionnaire was evaluated to detect moderate-to-severe OSA in highway bus drivers by Firat et al.[32] The prevalence of moderate-to-severe OSA among the highway bus drivers was 54%. The sensitivity and specificity of a STOP-Bang score ≥3 as the cut-off to detect moderate-to-severe OSA were 87% and 49% respectively, whereas the positive and negative predictive values were 66% and 76% respectively. The DOR was 6.3 and area under the ROC was 0.68.

#### Predictive parameters of the STOP-Bang questionnaire in renal failure patients

In renal failure patients, the prevalence of moderate-to-severe OSA (RDI ≥15) and severe OSA (RDI ≥30) was 42% and 29% respectively.[33] The sensitivities for a STOP-Bang score ≥ 3 as the cut-off to detect moderate-to-severe OSA (RDI ≥15) and severe OSA (RDI ≥30) were 93.1% and 98% respectively. The corresponding negative predictive values were 86% and 97%. The specificity was 30% and 27%. The PPV was 49% and 35% respectively.[33]

#### Predictive performance of various STOP-Bang scores

The predictive parameters of the various STOP-Bang score cut-offs were analyzed in six studies (n = 2807)[18,19,21,27,29,30]. Data from four studies (n = 1980)[21,27,29,30] from the sleep clinic population, and two (n = 827)[18,19] from the surgical population were pooled separately (Table 4 & Fig 2). In the sleep clinic population, as the STOP-Bang score cut-off increased from 3 to 8, the specificity increased from 52% to 100%, and the PPV increased continuously from 93% to 100% for any OSA (AHI ≥5). Similarly, for moderate-to-severe OSA (AHI ≥15) the specificity increased from 32% to 100%, and the PPV increased from 73% to 95%. For severe OSA (AHI ≥30) the specificity increased from 23% to 100% and PPV increased from 48% to 86% (Table 4).

**Table 4:** Predictive parameters of the various STOP-Bang score cut-offs for the different AHI levels in sleep clinic and surgical population.

Sleep Clinic population					Surgical population				
STOP-Bang Score cut-offs	Sensitivity	Specificity	PPV	NPV	STOP-Bang Score cut-offs	Sensitivity	Specificity	PPV	NPV
**All OSA (AHI ≥5)**					**All OSA (AHI ≥5)**				
≥1	100	2	89	100	≥1	99	3	69	50
≥2	98	20	90	58	≥2	96	18	72	66
≥3	91	52	93	44	≥3	84	40	75	54
≥4	76	71	95	30	≥4	60	61	77	41
≥5	54	84	96	20	≥5	36	80	79	37
≥6	30	93	97	16	≥6	18	92	82	34
≥7	12	98	97	14	≥7 & 8	4	98	82	32
= 8	2	100	100	13					
**Moderate/ Severe OSA (AHI ≥15)**					**Moderate/ Severe OSA (AHI ≥15)**				
≥1	100	1	67	100	≥1	99	7	40	94
≥2	99	10	68	79	≥2	99	1	38	60
≥3	94	32	73	74	≥3	90	11	39	61
≥4	81	51	76	58	≥4	70	32	40	62
≥5	60	72	80	48	≥5	45	56	40	61
≥6	35	89	86	42	≥6	23	78	40	61
≥7	14	96	88	37	≥7 & 8	4	95	37	61
= 8	3	100	95	35					
**Severe OSA (AHI ≥30)**					**Severe OSA (AHI ≥30)**				
≥1	100	1	42	100	≥1	100	2	18	100
≥2	99	7	43	89	≥2	100	10	20	100
≥3	96	23	47	88	≥3	95	28	22	96
≥4	85	43	52	79	≥4	78	52	26	92
≥5	66	66	58	72	≥5	56	74	32	88
≥6	42	85	67	67	≥6	28	88	35	85
≥7	19	96	76	60	≥7 & 8	6	97	33	83
= 8	4	100	86	58					

In the surgical population, as the STOP-Bang score cut-off increased from 3 to ≥7, the specificity increased from 40% to 98% and the PPV increased from 75% to 82% for any OSA (AHI ≥5). Similarly, for moderate-to-severe OSA (AHI ≥15) the specificity increased from 11% to 95%, but the PPV decreased from 39% to 37%. For severe OSA (AHI ≥30) the specificity increased from 28% to 97% and PPV increased from 22% to 33% (Table 4).

#### Association between STOP-Bang scores and predictive probability

**Fig 7:**
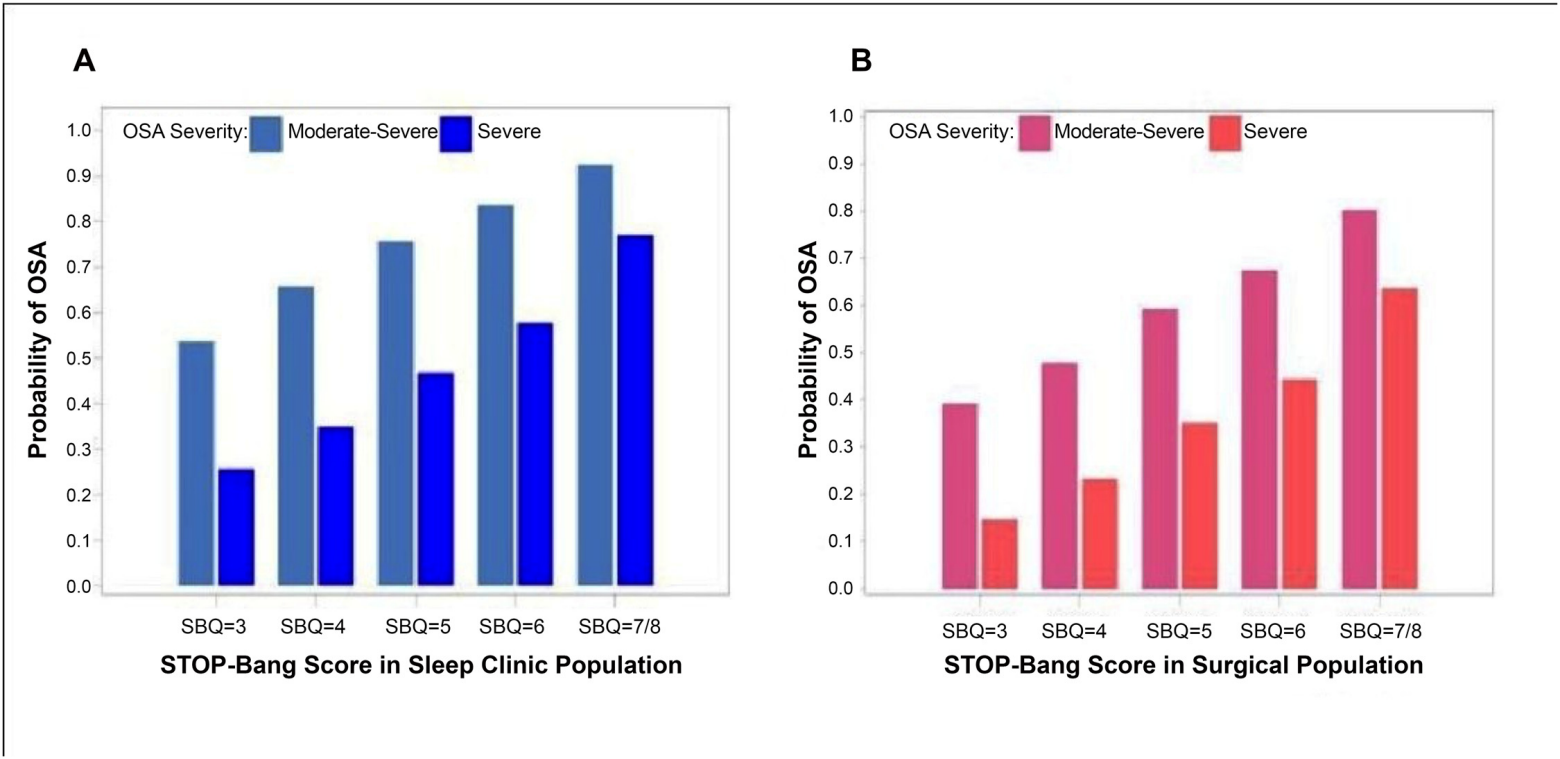
The relation between the various STOP-Bang scores and OSA probability. OSA—obstructive sleep apnea, AHI—apnea-hypopnea index, SBQ—STOP-Bang Questionnaire.

A meta-analysis was carried out in five studies (n = 2792),[18,21,29,30,51] three studies in sleep clinic patients (n = 1852)[21,29,30] and two studies in surgical patients, (n = 957)[18,51]. The relationship between the predictive probabilities of moderate-to-severe or severe OSA and STOP-Bang scores is illustrated in Fig 7. In both sleep clinic (Panel A; n = 1852) and surgical patients (Panel B; n = 957), the probability of moderate-to-severe OSA, or severe OSA increased as the STOP-Bang score increased from 3 to 7/ 8. With higher scores, there is a more profound increase in the probability of severe OSA compared to moderate OSA.

## Discussion

This review shows that the STOP-Bang questionnaire with a score ≥3 as the cut-off consistently demonstrates a high sensitivity to detect OSA in different patient populations; 94%(92–95) to detect moderate-to-severe OSA in sleep clinic patients and 91%(87–93) in surgical patients. The specificity at the same cut-off is modest, ranging from 34% in Sleep Clinic population to 32% in surgical population. As the STOP-Bang score increases, the probability of moderate and severe OSA increases. When the STOP-Bang score was 7 or 8, the probability of severe OSA was 75% in the sleep clinic population and 65% in the surgical population.

Given the relatively high prevalence of undiagnosed and untreated OSA[1,6,7] and its associated cardiovascular, respiratory, and neurocognitive morbidities[52–57], a simple and effective OSA screening tool is essential. This approach is important to perioperative care team, as often there is insufficient time to complete a preoperative assessment of OSA[58] with the standard diagnostic approach. The STOP-Bang questionnaire can fulfill this need given that it is a short, practical and straightforward test. The questionnaire can be completed within 1–2 minutes with very high response rates of 90–100%.[17]

### Utilization of the STOP-Bang questionnaire in the sleep clinic population

Since patients are referred to the sleep clinic for a suspicion of sleep related disorders, the prevalence of OSA is high in this population. The high sensitivity and NPV with a STOP-Bang score ≥3 as the cut-off can help sleep clinicians exclude patients with very little chance of moderate-to-severe OSA. On the other hand, a patient with a high score (≥5) on the STOP-Bang questionnaire has a high probability of severe OSA. These patients warrant expedited diagnosis and treatment. With the STOP-Bang questionnaire, sleep clinicians can prioritize their patients and efficiently allocate their limited resources.

### Utilization of the STOP-Bang questionnaire in the surgical population

OSA is prevalent in surgical populations and is considered to be an independent risk factor for perioperative complications in non-cardiac surgeries.[52–57] Further, OSA is associated with the occurrence of major adverse cardiovascular and cerebrovascular events, repeated revascularization, angina, and atrial fibrillation following coronary artery bypass grafting (CABG).[59] Mutter et al. have shown that surgical patients with a diagnosis of OSA and continuous positive airway pressure (CPAP) prescription had lower rates of cardiovascular complications.[60] Further, patients with OSA who are not treated with CPAP preoperatively are at increased risks for cardiopulmonary complications after general and vascular surgery.[61] Therefore, it is important to identify patients at high-risk of having moderate-to-severe OSA preoperatively. However, the short time interval between the preoperative clinic visit and scheduled surgery date, lack of willingness from patients to undergo preoperative PSG and potentially long wait times for a sleep clinic appointment may hinder diagnosing OSA prior to surgery. By incorporating the STOP-Bang questionnaire into preoperative clinic practice, surgical patients can be risk stratified for OSA severity using the score. A STOP-Bang score of 0–2 has a high negative predictive value for assessing the likelihood of moderate or severe OSA, which can be used to mitigate the need for PSG. Patients with a high score on the STOP-Bang questionnaire (≥5) have a high probability of having moderate-to-severe OSA. Depending on the co-morbidities and type of surgery, they may need referral to a sleep clinic for further investigation before surgery or be treated as an OSA patient perioperatively. Being able to predict moderate-to-severe or severe OSA in the perioperative setting is clinically relevant so that clinicians can take the appropriate steps in mitigating the risk of perioperative complications associated with OSA (e.g. changes in anesthetic care, careful titration of opioids, CPAP administration and postoperative monitoring).

In a retrospective study, Prockzco et al.[48] compared the outcomes of patients undergoing bariatric surgery who had undergone preoperative PSG and were on CPAP therapy to those considered high risk for OSA based on STOP-Bang score ≥3 without preoperative PSG. Patients with a STOP-Bang score ≥3 had higher postoperative complications and an increased length of stay (LOS) compared to patients with OSA using CPAP therapy perioperatively and compared to patients with a STOP-Bang score 0–2. This study was in line with others who found that patients with a STOP-Bang score ≥3 versus 0–2 had higher postoperative complications and longer LOS.[36][42] In a preoperative setting, a high STOP-Bang score may help in risk stratification and obviate the need for a PSG [62,63]. Moreover, perioperative CPAP therapy may reduce hospital LOS.[64] Therefore, identifying and treating patients at high risk for moderate or severe OSA may help to potentially avoid perioperative complication. Further research is needed in this area.

The variation in the predictive parameters among the different populations may be due to the difference in sample sizes, age and gender discrepancies of the recruited patients, differences in associated co-morbidities, or cultural / racial differences. In a study by Kunisaki et al[50], a STOP-Bang score ≥3 showed a high sensitivity of 99%, but a low specificity of 5%, which may be due to the predominantly older male population. The specific combination of predictive factors in the STOP-Bang questionnaire may improve its specificity. For patients with a STOP ≥2, male gender, a BMI >35 kg/m^2^ and a neck circumference >40 cm were more predictive of OSA than age [65]. The specificity of the STOP-Bang questionnaire may be improved by the addition of serum bicarbonate levels.[66] Most of the studies in our meta-analysis were from sleep clinic population, where the prevalence of OSA is higher. Further studies are required in a variety of medical, surgical, and general populations.

This systematic review and meta-analysis has some limitations. One of the factors contributing to the moderate to high heterogeneity is the variability of the target populations among the different studies. In an effort to unify the subject populations, all studies were divided into major groups: sleep clinic, surgical and general populations. The other reason for the heterogeneity may be variation in the prevalence of OSA in the different populations. Also, there was a paucity of validation studies in surgical patients. Nonetheless, we used the random effects method, which is more suitable when heterogeneity exists. The other limitation is that a non-English study was excluded even though it showed high sensitivity for the STOP-Bang questionnaire.[40] There is significant correlation between sensitivity and specificity with clinical screening tools, and our statistical approach does not account for overestimation of overall diagnostic test accuracy related to interpretation of each measure individually. More advanced methods utilizing bivariate or Bayesian frameworks may be necessary to address this limitation. Although DOR provides a combined measure across both sensitivity and specificity, it may significantly underestimate the confidence intervals. Despite these limitations, our systematic review and meta-analysis provides the interpretation of the available literature on the STOP-Bang questionnaire as a screening tool in OSA patients.

In summary, the STOP-Bang questionnaire has been validated to be an excellent screening tool for OSA in sleep clinic and surgical population. The probability of moderate and severe OSA steadily increases with higher STOP-Bang scores. The high negative predictive value of the STOP-Bang questionnaire may indicate that patients are unlikely to have moderate-to-severe OSA. These characteristics make the STOP-Bang questionnaire a useful clinical tool to identify patients at high risk of OSA and can facilitate the diagnosis and treatment of unrecognized OSA.

## Supporting Information

S1 Appendix Excluded studies and reasons for exclusion. (DOC)

S2 Appendix Appraisal of the included studies based on criteria for internal validity. (DOC)

S3 Appendix Appraisal of the included studies based on criteria for external validity. (DOC)

S1 Table Tables describing 2x2 contingency values and predictive parameter of individual studies for all OSA (AHI ≥5), moderate to severe (AHI ≥15) and severe OSA (AHI ≥30). (DOC)

## Abbreviations

AHI: Apnea/Hypopnea Index
AUC: Area under the curve
BMI: Body mass index
CI: Confidence Interval
CPAP: Continuous Positive Airway Pressure
CVS: Cardiovascular system
DM: Diabetes Mellitus
DOR: Diagnostic Odds Ratio
EDS: Excessive daytime sleepiness
EGD: Esophagoscopy
FN: False Negative
FP: False Positive
Lab: Laboratory
LOS: Length of Hospital stay
n: number of patient
NA: Not available
Neck cir: Neck circumference
NPV: Negative Predictive value
NS: Not Significant
OR: Odds Ratio
OSA: Obstructive Sleep Apnea
Postop Cx: Postoperative Complication
PPV: Positive Predictive Value
PRISMA: Preferred Reporting Items for Systematic Reviews and Meta-analysis
PSG: Polysomnogram
RDI: Respiratory Disturbance Index
ROC: Receiver Operating Characteristic
SB: STOP-Bang questionnaire
SPO_2_: Hemoglobin oxygen saturation
SROC: Summary of receiver operating characteristic curve
TN: True Negative
TP: True Positive
