# Supplementary material for: Validation of the STOP-Bang Questionnaire as a Screening Tool for Obstructive Sleep Apnea among Different Populations: A Systematic Review and Meta-Analysis
Source: PLoS One. 2015 Dec 14;10(12):e0143697. doi: 10.1371/journal.pone.0143697 (PMC4678295; doi:10.1371/journal.pone.0143697)
Supplement: S1 Appendix — (DOC) [file pone.0143697.s001.doc]

**S1 Appendix - Excluded studies**

| **Study ID** | **Use of STOP-Bang (SB)** | **n** | **High risk STOP-Bang**  **≥3** | **Low risk STOP-Bang**  **0-2** | **Results and reasons for exclusion** |
| --- | --- | --- | --- | --- | --- |
| ^[35]^Cote^2010^ | To predict hypoxemia & airway maneuvers in patients undergoing endoscopic procedures | 231 | 100 | 131 | Prevalence of STOP-Bang: 43.3%  Hypoxemia SB ≥3 vs. 0-2: 12 vs. 5.2%, P<0.0001  Airway maneuver SB ≥3 vs. 0-2: 20 vs. 6.1%, P<0.0001 |
| ^[36]^Vasu^2010^ | High risk STOP-Bang questionnaire predicts  Postop Cx | 135 | 56 | 79 | Postop Cx SB ≥3 vs. 0-2: 19.6 vs. 1.3%, P<0.001 |
| ^[37]^Alam^2012^ | Screening of OSA in patients with severe mental illness | 100 | 69 | 31 | High risk for OSA 69% |
| ^[38]^Coelho^2012^ | To identify OSA in Brazilian population during epidemiological study | 743 | 398 | 345 | Sensitivity AHI >15 – 83.8%; AHI >30 – 89.8%  Negative predictive value AHI >15: 87.5%; AHI >30: 98.26%  Inadequate information |
| ^[39]^Kim^2012^ | To predict apnea-hypopnea under Propofol sedation during spinal anesthesia | 34 | 15 | 14 | Severe apnea-hypopnea occurred more frequently in patients with higher SB scores.  SB ≥3 vs. 0-2: 27.6 vs. 6.9%, P=0.03 |
| ^[40]^Acar^2013^ | STOP-Bang questionnaire in Turkish population | 110 | 104 | 6 | Turkish language |
| ^[41]^Agrawal^2013^ | Association of STOP-Bang  & co-morbidities | 204 | 51 | 153 | STOP-Bang ≥3 vs. 0-2  CVS risk factors: 57 vs. 11.7%, P<0.001  Asthma & COPD: 14 vs. 3.8%, P=0.003  DM 22 vs. 5.2%, P<0.001  Hypothyroidism 6 vs. 1.9%, P<0.001 |
| ^[42]^Chia^2013^ | To predict postoperative  ICU admission | 5432 | 338 ICU admission |  | Odd ratios for ICU admission were 2.2 (P=0.037), 3.2 (P=0.017) & 5.1 (P=0.002) for STOP-Bang score of 4, 5 & 6. |
| ^[43]^Lockhart^2013^ | To predict postoperative mortality | 14,962 | 6226 | 8736 | 30 day postoperative mortality- No significant difference  1year mortality: SB ≥3 vs. 0-2: 7.45 vs.4.13%, P<0.0001 |
| ^[44]^Ozoh^2013^ | To predict OSA in commercial drivers | 500 | 244 | 256 | OSA high risk n=244 (48.8%)  Excessive daytime sleepiness (EDS): 14.4%  Positive correlation between high risk OSA and EDS |
| ^[45]^Pereira ^2013^ | To predict Postop Cx in PACU | 340 | 179 | 161 | STOP-Bang ≥3 vs. 0-2  Frequent mild/moderate hypoxemia 9 vs. 3%, P=0.012  Higher incidence of residual NMB 20 vs. 16%, P=0.035  Longer hospital stay 5 vs. 3days, P=0.01 |
| ^[46]^Acar^2014^ | STOP-Bang questionnaire to predict the difficulty intubation | 200 | 83 | 117 | Difficulty Intubation prevalence  STOP-Bang ≥3 vs. 0-2: 13.3% vs. 2.6%, P=0.004 |
| ^[47]^Mehta^2014^ | To predict CVS events during Propofol sedation for EGD/colonoscopy | 243 | 120 | 123 | Prevalence of STOP-Bang: 48.1%  Hypoxemia SB ≥3 vs. 0-2 11.2 vs. 16.9%, P=0.2  Hypotension SB ≥3 vs. 0-2 10.4 vs. 5.9%, P=0.21 |
| ^[48]^Proczko^2014^ | STOP-Bang questionnaire on patient outcome & LOS | 594 | 182 | 412 | STOP-Bang ≥3 vs. 0-2  LOS: 4.1 vs. 2.5days, P<0.0001  Pneumonia: 17 vs. 4, P<0.0001  Re-intubation: 7vs. 0, P<0.0098  ICU admission: 2vs. 0, P<0.00873  Death: 2vs. 0, P=0.3807 |
| ^[49]^Kulkarni^2014^ | Screening OSA in general surgery patients | 367 | 237 | 130 | Inadequate methodology and data |
| ^[50]^Kunisaki^2014^ | STOP-Bang questionnaire Veterans affair hospital | 1196 | RDI >15 =799 | RDI <15 =371 | RDI by Watch PAT 200  No gold standard test performed |
