# Supplementary material for: Validation of the STOP-Bang Questionnaire as a Screening Tool for Obstructive Sleep Apnea among Different Populations: A Systematic Review and Meta-Analysis
Source: PLoS One. 2015 Dec 14;10(12):e0143697. doi: 10.1371/journal.pone.0143697 (PMC4678295; doi:10.1371/journal.pone.0143697)
Supplement: S2 Appendix — (DOC) [file pone.0143697.s002.doc]

**S2 Appendix - Appraisal of the included studies based on criteria for internal** validity

| **Internal Criteria** | **Valid reference standard** | | **Definition of the disease based on reference standard** | **Blind execution of index test and reference test** | **Index test interpreted independently of clinical information** | **Study design** |
| --- | --- | --- | --- | --- | --- | --- |
| **Definition** | **Laboratory PSG (Lab PSG) or**  **Portable PSG (Port PSG)** | | **OSA diagnosed based on the PSG results (F)** | **PSG readings blinded to the questionnaire results and vice versa (F)** | **The questionnaire interpreted independently of clinical information (F)** | **Prospective or Retrospective** |
| Sleep Clinic population | | | | | | |
| ^[20]^Ong^2010^ | | Lab PSG | F | F | F | Prospective |
| ^[21]^Farney^2011^ | | Lab PSG | F | F | F | Retrospective |
| ^[22]^El-Sayed^2012^ | | Lab PSG | F | F | F | Prospective |
| ^[23]^Yu^2012^ | | Lab PSG | F | F | F | Prospective |
| ^[24]^Boynton ^2013^ | | Lab PSG | F | F | F | Prospective |
| ^[25]^Pereira^2013^ | | Lab PSG | F | F | F | Prospective |
| ^[26]^Vana ^2013^ | | Lab PSG | F | F | F | Prospective |
| ^[27]^Cowan^2014^ | | Port PSG | F | F | F | Prospective |
| ^[28]^Ha^2014^ | | Lab PSG | F | F | F | Prospective |
| ^[29]^Luo ^2014^ | | Lab PSG | F | F | F | Prospective |
| ^[30]^Reis^2015^ | | Lab/Port PSG | F | F | F | Prospective |
| Surgical population | | | | | | |
| ^[17]^Chung^2008^ | | Lab PSG | F | F | F | Prospective |
| ^[18]^Chung^2012^ | | Lab/Port PSG | F | F | F | Prospective |
| ^[19]^Nunes^2014^ | | Lab PSG | F | F | F | Prospective |
| General population | | | | | | |
| ^[31]^Silva ^2011^ | | Port PSG | F | F | F | Retrospective |
| Highway bus drivers | | | | | | |
| ^[32]^Firat^2012^ | | Lab PSG | F | F | F | Prospective |
| Renal failure patients | | | | | | |
| ^[33]^Nicholl^2013^ | | Port PSG | F | F | F | Prospective |
