# Supplementary material for: Validation of the STOP-Bang Questionnaire as a Screening Tool for Obstructive Sleep Apnea among Different Populations: A Systematic Review and Meta-Analysis
Source: PLoS One. 2015 Dec 14;10(12):e0143697. doi: 10.1371/journal.pone.0143697 (PMC4678295; doi:10.1371/journal.pone.0143697)
Supplement: S3 Appendix — (DOC) [file pone.0143697.s003.doc]

**S3 Appendix** - Appraisal of the included studies based on criteria for external validity

| **External Criteria** | **Spectrum of diseases** | | | **Settings** | **Previous screening** | | **Demographic information** | **Explication of cutoff point of index test** | **Percentage missing** | **Missing data management** | | **Subject selection for reference test** |
| --- | --- | --- | --- | --- | --- | --- | --- | --- | --- | --- | --- | --- |
| **Definition** | **Inclusion and exclusion criteria mentioned (F)** | | | **Enough information to identify setting (F)** | **No pre-screening before application of the questionnaire (F)** | | **Age, gender, BMI data provided (F)** | **Results presented for AHI ≥5 or RDI≥15 (F)** | **Percentage missing mentioned (F)** | **Analysis of missing data for basic characteristics (F)** | | **All subjects were invited or randomly selected to do PSG (F)** |
| Sleep clinic Population | | | | | | | | | | | | |
| ^[20]^Ong^2010^ | | | F | F | F | F | | F | F | F | F | |
| ^[21]^Farney^2011^ | | | F | F | F | F | | F | F | F | F | |
| ^[22]^El-Sayed^2012^ | | | F | F | F | F | | F | F | F | F | |
| ^[23]^Yu ^2012^ | | | F | F | F | F | | F | F | F | F | |
| ^[24]^Boynton^2013^ | | | F | F | F | F | | F | F | F | F | |
| ^[25]^Pereira^2013^ | | | F | F | F | F | | F | F | F | F | |
| ^[26]^Vana^2013^ | | | F | F | F | F | | F | F | F | F | |
| ^[27]^Cowan^2014^ | | | F | F | F | F | | F | F | F | F | |
| ^[28]^Ha^2014^ | | | F | F | F | F | | F | F | F | F | |
| ^[29]^Luo^2014^ | | | F | F | F | F | | F | F | F | F | |
| ^[30]^Reis ^2015^ | | | F | F | F | F | | F | F | F | F | |
| Surgical population | | | | | | | | | | | | |
| ^[17]^Chung^2008^ | | | F | F | F | F | | F | F | F | F | |
| ^[18]^Chung^2012^ | | | F | F | F | F | | F | F | F | F | |
| ^[19]^Nunes^2014^ | | | F | F | F | F | | F | F | F | F | |
| General population and Renal patients | | | | | | | | | | | | |
| ^[31]^Silva ^2011^ | | | F | F | F | F | | F | F | F | F | |
| Highway bus drivers | | | | | | | | | | | | |
| ^[32]^Firat^2012^ | | F | | F | F | N | | F | F | F | F | |
| Renal failure patients | | | | | | | | | | | | |
| ^[33]^Nicholl^2013^ | | F | | F | F | F | | F | F | F | F | |
