# Supplementary material for: Validation of the STOP-Bang Questionnaire as a Screening Tool for Obstructive Sleep Apnea among Different Populations: A Systematic Review and Meta-Analysis
Source: PLoS One. 2015 Dec 14;10(12):e0143697. doi: 10.1371/journal.pone.0143697 (PMC4678295; doi:10.1371/journal.pone.0143697)
Supplement: S1 Table — (DOC) [file pone.0143697.s004.doc]

| **Author** | **True Positive** | **False Positive** | **False Negative** | **True Negative** | **Sensitivity (95% Confidence Interval)** | **Specificity (95% Confidence Interval)** |
| --- | --- | --- | --- | --- | --- | --- |
| Ong 2010 | 207 | 34 | 37 | 36 | 0.85 [0.80, 0.89] | 0.51 [0.39, 0.64] |
| Farney 2011 | 1140 | 63 | 134 | 87 | 0.89 [0.88, 0.91] | 0.58 [0.50, 0.66] |
| El-Sayed 2012 | 199 | 22 | 5 | 8 | 0.98 [0.94, 0.99] | 0.27 [0.12, 0.46] |
| Yu 2012 | 78 | 11 | 13 | 12 | 0.86 [0.77, 0.92] | 0.52 [0.31, 0.73] |
| Boynton 2013 | 139 | 26 | 30 | 24 | 0.82 [0.76, 0.88] | 0.48 [0.34, 0.63] |
| Pereire 2013 | 104 | 7 | 12 | 5 | 0.90 [0.83, 0.95] | 0.42 [0.15, 0.72] |
| Vana 2013 | 30 | 10 | 2 | 5 | 0.94 [0.79, 0.99] | 0.33 [0.12, 0.62] |
| Cowan 2014 | 92 | 22 | 5 | 10 | 0.95 [0.88, 0.98] | 0.31 [0.16, 0.50] |
| Ha 2014 | 90 | 12 | 21 | 16 | 0.81 [0.73, 0.88] | 0.57 [0.37, 0.76] |
| Luo 2014 | 186 | 8 | 10 | 8 | 0.95 [0.91, 0.98] | 0.50 [0.25, 0.75] |
| Reis 2015 | 157 | 24 | 11 | 23 | 0.93 [0.89, 0.97] | 0.49 [0.34, 0.64] |

|  | Polysomnography Positive | Polysomnography Negative |
| --- | --- | --- |
| STOP-Bang Positive | True Positive | False Positive |
| STOP-Bang Negative | False Negative | True Negative |

S1 Table – Tables describing 2x2 contingency values and predictive parameter of individual studies for all OSA (AHI ≥5), moderate to severe (AHI ≥15) and severe OSA (AHI ≥30)

S1A: Description of 2X2 contingency table

S1B: 2X2 contingency table for Sleep Clinic Population - All OSA or AHI ≥5

S1C: 2X2 contingency table for Surgical Population - All OSA or AHI ≥5

| **Author** | **True Positive** | **False Positive** | **False Negative** | **True Negative** | **Sensitivity (95% Confidence Interval)** | **Specificity (95% Confidence Interval)** |
| --- | --- | --- | --- | --- | --- | --- |
| Chung 2008 | 102 | 24 | 20 | 31 | 0.84 [0.76, 0.90] | 0.56 [0.42, 0.70] |
| Chung 2012 | 429 | 141 | 81 | 95 | 0.84 [0.81, 0.87] | 0.40 [0.34, 0.47] |

S1D: 2X2 contingency table for Sleep Clinic Population - Moderate to Severe OSA or AHI ≥15

| **Author** | **True Positive** | **False Positive** | **False Negative** | **True Negative** | **Sensitivity (95% Confidence Interval)** | **Specificity (95% Confidence Interval)** |
| --- | --- | --- | --- | --- | --- | --- |
| Ong 2010 | 151 | 90 | 15 | 58 | 0.91 [0.86, 0.95] | 0.39 [0.31, 0.48] |
| Farney 2011 | 895 | 307 | 64 | 158 | 0.93 [0.92, 0.95] | 0.34 [0.30, 0.38] |
| El-Sayed 2012 | 198 | 30 | 5 | 1 | 0.98 [0.94, 0.99] | 0.03 [0.00, 0.17] |
| Yu 2012 | 62 | 27 | 5 | 20 | 0.93 [0.83, 0.98] | 0.43 [0.28, 0.58] |
| Boynton 2013 | 96 | 69 | 7 | 47 | 0.93 [0.86, 0.97] | 0.41 [0.32, 0.50] |
| Pereire 2013 | 82 | 29 | 6 | 11 | 0.93 [0.86, 0.97] | 0.28 [0.15, 0.44] |
| Vana 2013 | 18 | 22 | 1 | 6 | 0.95 [0.74, 1.00] | 0.21 [0.08, 0.41] |
| Cowan 2014 | 56 | 56 | 0 | 17 | 1.00 [0.94, 1.00] | 0.23 [0.14, 0.35] |
| Ha 2014 | 72 | 30 | 12 | 25 | 0.86 [0.76, 0.92] | 0.45 [0.32, 0.59] |
| Luo 2014 | 164 | 30 | 6 | 12 | 0.96 [0.92, 0.99] | 0.29 [0.16, 0.45] |
| Reis 2015 | 113 | 68 | 5 | 29 | 0.96 [0.90, 0.99] | 0.30 [0.21, 0.40] |

S1E: 2X2 contingency table for Surgical Population - Moderate to Severe OSA or AHI ≥15

| **Author** | **True Positive** | **False Positive** | **False Negative** | **True Negative** | **Sensitivity (95% Confidence Interval)** | **Specificity (95% Confidence Interval)** |
| --- | --- | --- | --- | --- | --- | --- |
| Chung 2008 | 65 | 61 | 5 | 46 | 0.93 [0.84, 0.98] | 0.43 [0.33, 0.53] |
| Chung 2012 | 256 | 314 | 29 | 147 | 0.90 [0.86, 0.93] | 0.32 [0.28, 0.36] |

S1F: 2X2 contingency table for **Other Miscellaneous studies -** Moderate to Severe OSA or AHI ≥15

| **Author** | **True Positive** | **False Positive** | **False Negative** | **True Negative** | **Sensitivity (95% Confidence Interval)** | **Specificity (95% Confidence Interval)** |
| --- | --- | --- | --- | --- | --- | --- |
| Firat 2012 | 40 | 20 | 6 | 19 | 0.87 [0.74, 0.95] | 0.49 [0.32, 0.65] |
| Nicholl 2013 | 68 | 69 | 5 | 30 | 0.93 [0.85, 0.98] | 0.30 [0.21, 0.40] |
| Silva 2011 | 534 | 2920 | 69 | 1247 | 0.89 [0.86, 0.91] | 0.30 [0.29, 0.31] |

S1G: 2X2 contingency table for Sleep Clinic Population - Severe OSA or AHI ≥30

| **Author** | **True Positive** | **False Positive** | **False Negative** | **True Negative** | **Sensitivity (95% Confidence Interval)** | **Specificity (95% Confidence Interval)** |
| --- | --- | --- | --- | --- | --- | --- |
| Ong 2010 | 108 | 133 | 5 | 68 | 0.96 [0.90, 0.99] | 0.34 [0.27, 0.41] |
| Farney 2011 | 555 | 647 | 25 | 197 | 0.96 [0.94, 0.97] | 0.23 [0.21, 0.26] |
| El-Sayed 2012 | 146 | 78 | 2 | 4 | 0.99 [0.95, 1.00] | 0.05 [0.01, 0.12] |
| Yu 2012 | 46 | 43 | 0 | 25 | 1.00 [0.92, 1.00] | 0.37 [0.25, 0.49] |
| Boynton 2013 | 60 | 105 | 2 | 52 | 0.97 [0.89, 1.00] | 0.33 [0.26, 0.41] |
| Pereire 2013 | 54 | 57 | 2 | 15 | 0.96 [0.88, 1.00] | 0.21 [0.12, 0.32] |
| Ha 2014 | 44 | 58 | 7 | 30 | 0.86 [0.74, 0.94] | 0.34 [0.24, 0.45] |
| Luo 2014 | 125 | 69 | 3 | 15 | 0.98 [0.93, 1.00] | 0.18 [0.10, 0.28] |
| Reis 2015 | 61 | 120 | 1 | 34 | 0.98 [0.91, 1.00] | 0.22 [0.16, 0.29] |

S1H: 2X2 contingency table for Surgical Population - Severe OSA or AHI ≥30

| **Author** | **True Positive** | **False Positive** | **False Negative** | **True Negative** | **Sensitivity (95% Confidence Interval)** | **Specificity (95% Confidence Interval)** |
| --- | --- | --- | --- | --- | --- | --- |
| Chung 2008 | 39 | 87 | 0 | 51 | 1.00 [0.91, 1.00] | 0.37 [0.29, 0.46] |
| Chung 2012 | 127 | 443 | 7 | 169 | 0.95 [0.90, 0.98] | 0.28 [0.24, 0.31] |

S1I: 2X2 contingency table for **Other Miscellaneous studies -** Severe OSA or AHI ≥30

| **Author** | **True Positive** | **False Positive** | **False Negative** | **True Negative** | **Sensitivity (95% Confidence Interval)** | **Specificity (95% Confidence Interval)** |
| --- | --- | --- | --- | --- | --- | --- |
| Nicholl 2013 | 49 | 89 | 1 | 33 | 0.98 [0.89, 1.00] | 0.27 [0.19, 0.36] |
| Silva 2011 | 320 | 3134 | 25 | 1291 | 0.93 [0.89, 0.95] | 0.29 [0.28, 0.31] |

S1J: Ong et al. 2010; Sleep Clinic Population - All OSA or AHI ≥5

| **Statistic** | **Formula** | **Value** | **95% CI** |
| --- | --- | --- | --- |
| Sensitivity | 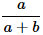 | 84.84% | 79.71% to 89.09% |
| Specificity | 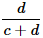 | 51.43 % | 39.17% to 63.56% |
| Positive Likelihood Ratio | 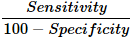 | 1.75 | 1.36 to 2.24 |
| Negative Likelihood Ratio | 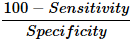 | 0.29 | 0.20 to 0.43 |
| Disease prevalence | 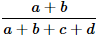 | 77.71% (*) | 72.69% to 82.19% |
| Positive Predictive Value | 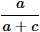 | 85.89% (*) | 80.85% to 90.03% |
| Negative Predictive Value | 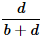 | 49.32 % (*) | 37.40% to 61.28% |

S1K: Farney et al. 2011; Sleep Clinic Population - All OSA or AHI ≥5

| **Statistic** | **Formula** | **Value** | **95% CI** |
| --- | --- | --- | --- |
| Sensitivity | 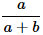 | 89.48% | 87.67% to 91.11% |
| Specificity | 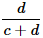 | 58.00 % | 49.68% to 66.00% |
| Positive Likelihood Ratio | 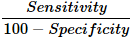 | 2.13 | 1.76 to 2.57 |
| Negative Likelihood Ratio | 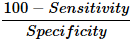 | 0.18 | 0.15 to 0.22 |
| Disease prevalence | 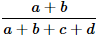 | 89.47% (*) | 87.75% to 91.01% |
| Positive Predictive Value | 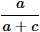 | 94.76% (*) | 93.35% to 95.95% |
| Negative Predictive Value | 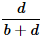 | 39.37 % (*) | 32.88% to 46.14% |

S1L: El-Sayed et al. 2012; Sleep Clinic Population - All OSA or AHI ≥5

| **Statistic** | **Formula** | **Value** | **95% CI** |
| --- | --- | --- | --- |
| Sensitivity | 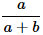 | 97.55% | 94.37% to 99.20% |
| Specificity | 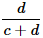 | 26.67 % | 12.28% to 45.89% |
| Positive Likelihood Ratio | 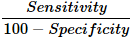 | 1.33 | 1.07 to 1.65 |
| Negative Likelihood Ratio | 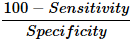 | 0.09 | 0.03 to 0.26 |
| Disease prevalence | 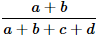 | 87.18% (*) | 82.21% to 91.18% |
| Positive Predictive Value | 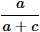 | 90.05% (*) | 85.32% to 93.66% |
| Negative Predictive Value | 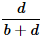 | 61.54 % (*) | 31.58% to 86.14% |

S1M: Yu et al. 2012; Sleep Clinic Population - All OSA or AHI ≥5

| **Statistic** | **Formula** | **Value** | **95% CI** |
| --- | --- | --- | --- |
| Sensitivity | 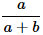 | 85.71% | 76.81% to 92.17% |
| Specificity | 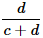 | 52.17 % | 30.59% to 73.18% |
| Positive Likelihood Ratio | 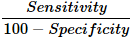 | 1.79 | 1.16 to 2.77 |
| Negative Likelihood Ratio | 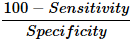 | 0.27 | 0.14 to 0.52 |
| Disease prevalence | 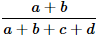 | 79.82% (*) | 71.28% to 86.76% |
| Positive Predictive Value | 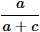 | 87.64% (*) | 78.96% to 93.67% |
| Negative Predictive Value | 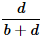 | 48.00 % (*) | 27.80% to 68.69% |

S1N: Boynton et al. 2013; Sleep Clinic Population - All OSA or AHI ≥5

| **Statistic** | **Formula** | **Value** | **95% CI** |
| --- | --- | --- | --- |
| Sensitivity | 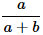 | 82.25% | 75.64% to 87.69% |
| Specificity | 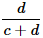 | 48.00 % | 33.66% to 62.58% |
| Positive Likelihood Ratio | 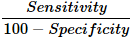 | 1.58 | 1.20 to 2.08 |
| Negative Likelihood Ratio | 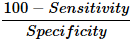 | 0.37 | 0.24 to 0.57 |
| Disease prevalence | 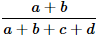 | 77.17% (*) | 71.03% to 82.55% |
| Positive Predictive Value | 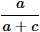 | 84.24% (*) | 77.77% to 89.44% |
| Negative Predictive Value | 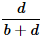 | 44.44 % (*) | 30.92% to 58.60% |

S1O: Pereira et al. 2013; Sleep Clinic Population - All OSA or AHI ≥5

| **Statistic** | **Formula** | **Value** | **95% CI** |
| --- | --- | --- | --- |
| Sensitivity | 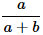 | 89.66% | 82.63% to 94.54% |
| Specificity | 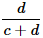 | 41.67 % | 15.17% to 72.33% |
| Positive Likelihood Ratio | 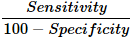 | 1.54 | 0.95 to 2.49 |
| Negative Likelihood Ratio | 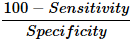 | 0.25 | 0.11 to 0.59 |
| Disease prevalence | 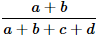 | 90.62% (*) | 84.20% to 95.06% |
| Positive Predictive Value | 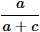 | 93.69% (*) | 87.44% to 97.43% |
| Negative Predictive Value | 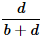 | 29.41 % (*) | 10.31% to 55.96% |

S1P: Vana et al. 2013; Sleep Clinic Population - All OSA or AHI ≥5

| **Statistic** | **Formula** | **Value** | **95% CI** |
| --- | --- | --- | --- |
| Sensitivity | 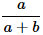 | 93.75% | 79.19% to 99.23% |
| Specificity | 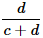 | 33.33 % | 11.82% to 61.62% |
| Positive Likelihood Ratio | 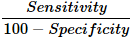 | 1.41 | 0.97 to 2.03 |
| Negative Likelihood Ratio | 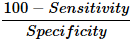 | 0.19 | 0.04 to 0.86 |
| Disease prevalence | 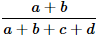 | 68.09% (*) | 52.88% to 80.91% |
| Positive Predictive Value | 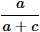 | 75.00% (*) | 58.80% to 87.31% |
| Negative Predictive Value | 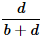 | 71.43 % (*) | 29.04% to 96.33% |

S1Q: Cowan et al. 2014; Sleep Clinic Population - All OSA or AHI ≥5

| **Statistic** | **Formula** | **Value** | **95% CI** |
| --- | --- | --- | --- |
| Sensitivity | 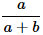 | 94.85% | 88.38% to 98.31% |
| Specificity | 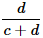 | 31.25 % | 16.12% to 50.01% |
| Positive Likelihood Ratio | 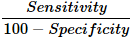 | 1.38 | 1.09 to 1.75 |
| Negative Likelihood Ratio | 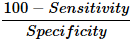 | 0.16 | 0.06 to 0.45 |
| Disease prevalence | 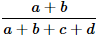 | 75.19% (*) | 66.82% to 82.37% |
| Positive Predictive Value | 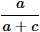 | 80.70% (*) | 72.25% to 87.49% |
| Negative Predictive Value | 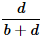 | 66.67 % (*) | 38.38% to 88.18% |

S1R: Ha et al. 2014; Sleep Clinic Population - All OSA or AHI ≥5

| **Statistic** | **Formula** | **Value** | **95% CI** |
| --- | --- | --- | --- |
| Sensitivity | 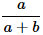 | 81.08% | 72.55% to 87.89% |
| Specificity | 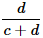 | 57.14 % | 37.18% to 75.54% |
| Positive Likelihood Ratio | 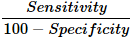 | 1.89 | 1.22 to 2.93 |
| Negative Likelihood Ratio | 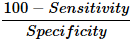 | 0.33 | 0.20 to 0.55 |
| Disease prevalence | 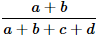 | 79.86% (*) | 72.22% to 86.18% |
| Positive Predictive Value | 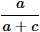 | 88.24% (*) | 80.35% to 93.77% |
| Negative Predictive Value | 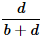 | 43.24 % (*) | 27.10% to 60.51% |

S1S: Luo et al. 2014; Sleep Clinic Population - All OSA or AHI ≥5

| **Statistic** | **Formula** | **Value** | **95% CI** |
| --- | --- | --- | --- |
| Sensitivity | 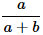 | 94.90% | 90.82% to 97.53% |
| Specificity | 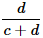 | 50.00 % | 24.65% to 75.35% |
| Positive Likelihood Ratio | 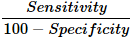 | 1.90 | 1.16 to 3.10 |
| Negative Likelihood Ratio | 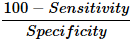 | 0.10 | 0.05 to 0.22 |
| Disease prevalence | 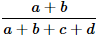 | 92.45% (*) | 88.03% to 95.62% |
| Positive Predictive Value | 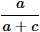 | 95.88% (*) | 92.04% to 98.20% |
| Negative Predictive Value | 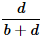 | 44.44 % (*) | 21.53% to 69.24% |

S1T: Reis et al. 2015; Sleep Clinic Population - All OSA or AHI ≥5

| **Statistic** | **Formula** | **Value** | **95% CI** |
| --- | --- | --- | --- |
| Sensitivity | 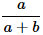 | 93.45% | 88.59% to 96.69% |
| Specificity | 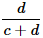 | 48.94 % | 34.08% to 63.94% |
| Positive Likelihood Ratio | 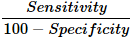 | 1.83 | 1.38 to 2.43 |
| Negative Likelihood Ratio | 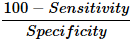 | 0.13 | 0.07 to 0.25 |
| Disease prevalence | 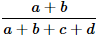 | 78.14% (*) | 72.01% to 83.47% |
| Positive Predictive Value | 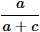 | 86.74% (*) | 80.92% to 91.32% |
| Negative Predictive Value | 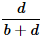 | 67.65 % (*) | 49.47% to 82.61% |

S1U: Chung et al. 2008; Surgical Population - All OSA or AHI ≥5

| **Statistic** | **Formula** | **Value** | **95% CI** |
| --- | --- | --- | --- |
| Sensitivity | 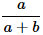 | 83.61% | 75.82% to 89.69% |
| Specificity | 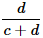 | 56.36 % | 42.32% to 69.70% |
| Positive Likelihood Ratio | 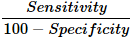 | 1.92 | 1.40 to 2.61 |
| Negative Likelihood Ratio | 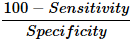 | 0.29 | 0.18 to 0.46 |
| Disease prevalence | 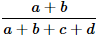 | 68.93% (*) | 61.55% to 75.66% |
| Positive Predictive Value | 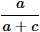 | 80.95% (*) | 73.00% to 87.40% |
| Negative Predictive Value | 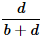 | 60.78 % (*) | 46.11% to 74.16% |

S1V: Chung et al. 2012; Surgical Population - All OSA or AHI ≥5

| **Statistic** | **Formula** | **Value** | **95% CI** |
| --- | --- | --- | --- |
| Sensitivity | 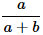 | 84.12% | 80.65% to 87.18% |
| Specificity | 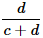 | 40.25 % | 33.94% to 46.81% |
| Positive Likelihood Ratio | 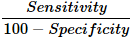 | 1.41 | 1.26 to 1.57 |
| Negative Likelihood Ratio | 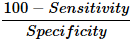 | 0.39 | 0.31 to 0.51 |
| Disease prevalence | 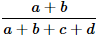 | 68.36% (*) | 64.89% to 71.69% |
| Positive Predictive Value | 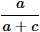 | 75.26% (*) | 71.51% to 78.75% |
| Negative Predictive Value | 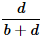 | 53.98 % (*) | 46.32% to 61.50% |

S1W: Ong et al. 2010; Sleep Clinic Population – Moderate to Severe OSA or AHI ≥15

| **Statistic** | **Formula** | **Value** | **95% CI** |
| --- | --- | --- | --- |
| Sensitivity | 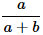 | 90.96% | 85.53% to 94.85% |
| Specificity | 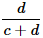 | 39.19 % | 31.28% to 47.54% |
| Positive Likelihood Ratio | 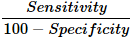 | 1.50 | 1.30 to 1.72 |
| Negative Likelihood Ratio | 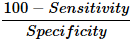 | 0.23 | 0.14 to 0.39 |
| Disease prevalence | 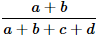 | 52.87% (*) | 47.18% to 58.50% |
| Positive Predictive Value | 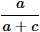 | 62.66% (*) | 56.22% to 68.78% |
| Negative Predictive Value | 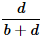 | 79.45 % (*) | 68.38% to 88.02% |

S1X: Farney et al. 2011; Sleep Clinic Population - Moderate to Severe OSA or AHI ≥15

| **Statistic** | **Formula** | **Value** | **95% CI** |
| --- | --- | --- | --- |
| Sensitivity | 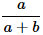 | 93.33% | 91.56% to 94.82% |
| Specificity | 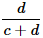 | 33.98 % | 29.68% to 38.48% |
| Positive Likelihood Ratio |  | 1.41 | 1.32 to 1.51 |
| Negative Likelihood Ratio |  | 0.20 | 0.15 to 0.26 |
| Disease prevalence |  | 67.35% (*) | 64.84% to 69.78% |
| Positive Predictive Value |  | 74.46% (*) | 71.89% to 76.90% |
| Negative Predictive Value |  | 71.17 % (*) | 64.73% to 77.04% |

S1Y: El-Sayed et al. 2012; Sleep Clinic Population - Moderate to Severe OSA or AHI ≥15

| **Statistic** | **Formula** | **Value** | **95% CI** |
| --- | --- | --- | --- |
| Sensitivity |  | 97.54% | 94.35% to 99.20% |
| Specificity |  | 3.23 % | 0.08% to 16.70% |
| Positive Likelihood Ratio |  | 1.01 | 0.94 to 1.08 |
| Negative Likelihood Ratio |  | 0.76 | 0.09 to 6.32 |
| Disease prevalence |  | 86.75% (*) | 81.73% to 90.82% |
| Positive Predictive Value |  | 86.84% (*) | 81.75% to 90.94% |
| Negative Predictive Value |  | 16.67 % (*) | 0.42% to 64.12% |

S1Z: Yu et al. 2012; Sleep Clinic Population - Moderate to Severe OSA or AHI ≥15

| **Statistic** | **Formula** | **Value** | **95% CI** |
| --- | --- | --- | --- |
| Sensitivity |  | 92.54% | 83.44% to 97.53% |
| Specificity |  | 42.55 % | 28.26% to 57.82% |
| Positive Likelihood Ratio |  | 1.61 | 1.25 to 2.08 |
| Negative Likelihood Ratio |  | 0.18 | 0.07 to 0.43 |
| Disease prevalence |  | 58.77% (*) | 49.17% to 67.91% |
| Positive Predictive Value |  | 69.66% (*) | 59.01% to 78.97% |
| Negative Predictive Value |  | 80.00 % (*) | 59.30% to 93.17% |

S1Aa: Boynton et al. 2013; Sleep Clinic Population - Moderate to Severe OSA or AHI ≥15

| **Statistic** | **Formula** | **Value** | **95% CI** |
| --- | --- | --- | --- |
| Sensitivity |  | 93.20% | 86.50% to 97.22% |
| Specificity |  | 40.52 % | 31.50% to 50.03% |
| Positive Likelihood Ratio |  | 1.57 | 1.34 to 1.84 |
| Negative Likelihood Ratio |  | 0.17 | 0.08 to 0.35 |
| Disease prevalence |  | 47.03% (*) | 40.27% to 53.87% |
| Positive Predictive Value |  | 58.18% (*) | 50.26% to 65.80% |
| Negative Predictive Value |  | 87.04 % (*) | 75.10% to 94.63% |

S1Ba: Pereira et al. 2013; Sleep Clinic Population - Moderate to Severe OSA or AHI ≥15

| **Statistic** | **Formula** | **Value** | **95% CI** |
| --- | --- | --- | --- |
| Sensitivity |  | 93.18% | 85.75% to 97.46% |
| Specificity |  | 27.50 % | 14.60% to 43.89% |
| Positive Likelihood Ratio |  | 1.29 | 1.05 to 1.57 |
| Negative Likelihood Ratio |  | 0.25 | 0.10 to 0.62 |
| Disease prevalence |  | 68.75% (*) | 59.96% to 76.65% |
| Positive Predictive Value |  | 73.87% (*) | 64.68% to 81.75% |
| Negative Predictive Value |  | 64.71 % (*) | 38.33% to 85.79% |

S1Ca: Vana et al. 2013; Sleep Clinic Population - Moderate to Severe OSA or AHI ≥15

| **Statistic** | **Formula** | **Value** | **95% CI** |
| --- | --- | --- | --- |
| Sensitivity |  | 94.74% | 73.97% to 99.87% |
| Specificity |  | 21.43 % | 8.30% to 40.95% |
| Positive Likelihood Ratio |  | 1.21 | 0.97 to 1.50 |
| Negative Likelihood Ratio |  | 0.25 | 0.03 to 1.88 |
| Disease prevalence |  | 40.43% (*) | 26.37% to 55.73% |
| Positive Predictive Value |  | 45.00% (*) | 29.26% to 61.51% |
| Negative Predictive Value |  | 85.71 % (*) | 42.13% to 99.64% |

S1Da: Cowan et al. 2014; Sleep Clinic Population - Moderate to Severe OSA or AHI ≥15

| **Statistic** | **Formula** | **Value** | **95% CI** |
| --- | --- | --- | --- |
| Sensitivity |  | 100.00% | 93.62% to 100.00% |
| Specificity |  | 23.29 % | 14.19% to 34.65% |
| Positive Likelihood Ratio |  | 1.30 | 1.15 to 1.48 |
| Negative Likelihood Ratio |  | 0.00 |  |
| Disease prevalence |  | 43.41% (*) | 34.71% to 52.42% |
| Positive Predictive Value |  | 50.00% (*) | 40.40% to 59.60% |
| Negative Predictive Value |  | 100.00 % (*) | 80.49% to 100.00% |

S1Ea: Ha et al. 2014; Sleep Clinic Population - Moderate to Severe OSA or AHI ≥15

| **Statistic** | **Formula** | **Value** | **95% CI** |
| --- | --- | --- | --- |
| Sensitivity |  | 85.71% | 76.38% to 92.39% |
| Specificity |  | 45.45 % | 31.97% to 59.45% |
| Positive Likelihood Ratio |  | 1.57 | 1.22 to 2.03 |
| Negative Likelihood Ratio |  | 0.31 | 0.17 to 0.57 |
| Disease prevalence |  | 60.43% (*) | 51.79% to 68.62% |
| Positive Predictive Value |  | 70.59% (*) | 60.75% to 79.20% |
| Negative Predictive Value |  | 67.57 % (*) | 50.21% to 81.99% |

S1Fa: Luo et al. 2014; Sleep Clinic Population - Moderate to Severe OSA or AHI ≥15

| **Statistic** | **Formula** | **Value** | **95% CI** |
| --- | --- | --- | --- |
| Sensitivity |  | 96.47% | 92.48% to 98.69% |
| Specificity |  | 28.57 % | 15.72% to 44.58% |
| Positive Likelihood Ratio |  | 1.35 | 1.11 to 1.64 |
| Negative Likelihood Ratio |  | 0.12 | 0.05 to 0.31 |
| Disease prevalence |  | 80.19% (*) | 74.18% to 85.33% |
| Positive Predictive Value |  | 84.54% (*) | 78.67% to 89.32% |
| Negative Predictive Value |  | 66.67 % (*) | 40.99% to 86.66% |

S1Ga: Reis et al. 2015; Sleep Clinic Population - Moderate to Severe OSA or AHI ≥15

| **Statistic** | **Formula** | **Value** | **95% CI** |
| --- | --- | --- | --- |
| Sensitivity |  | 95.76% | 90.39% to 98.61% |
| Specificity |  | 29.90 % | 21.02% to 40.04% |
| Positive Likelihood Ratio |  | 1.37 | 1.19 to 1.56 |
| Negative Likelihood Ratio |  | 0.14 | 0.06 to 0.35 |
| Disease prevalence |  | 54.88% (*) | 47.97% to 61.66% |
| Positive Predictive Value |  | 62.43% (*) | 54.94% to 69.51% |
| Negative Predictive Value |  | 85.29 % (*) | 68.94% to 95.05% |

S1Ha: Chung et al. 2008; Surgical Population - Moderate to Severe OSA or AHI ≥15

| **Statistic** | **Formula** | **Value** | **95% CI** |
| --- | --- | --- | --- |
| Sensitivity |  | 92.86% | 84.11% to 97.64% |
| Specificity |  | 42.99 % | 33.46% to 52.92% |
| Positive Likelihood Ratio |  | 1.63 | 1.36 to 1.94 |
| Negative Likelihood Ratio |  | 0.17 | 0.07 to 0.40 |
| Disease prevalence |  | 39.55% (*) | 32.29% to 47.16% |
| Positive Predictive Value |  | 51.59% (*) | 42.52% to 60.58% |
| Negative Predictive Value |  | 90.20 % (*) | 78.59% to 96.74% |

S1Ia: Chung et al. 2012; Surgical Population - Moderate to Severe OSA or AHI ≥15

| **Statistic** | **Formula** | **Value** | **95% CI** |
| --- | --- | --- | --- |
| Sensitivity |  | 89.82% | 85.71% to 93.08% |
| Specificity |  | 31.89 % | 27.65% to 36.36% |
| Positive Likelihood Ratio |  | 1.32 | 1.23 to 1.42 |
| Negative Likelihood Ratio |  | 0.32 | 0.22 to 0.46 |
| Disease prevalence |  | 38.20% (*) | 34.70% to 41.80% |
| Positive Predictive Value |  | 44.91% (*) | 40.78% to 49.10% |
| Negative Predictive Value |  | 83.52 % (*) | 77.20% to 88.68% |

S1Ja: Nunes et al. 2015; Sleep Clinic Population - Moderate to Severe OSA or AHI ≥15

| **Statistic** | **Formula** | **Value** | **95% CI** |
| --- | --- | --- | --- |
| Sensitivity |  | 92.31% | 79.13% to 98.38% |
| Specificity |  | 7.14 % | 1.50% to 19.48% |
| Positive Likelihood Ratio |  | 0.99 | 0.88 to 1.12 |
| Negative Likelihood Ratio |  | 1.08 | 0.23 to 5.02 |
| Disease prevalence |  | 48.15% (*) | 36.90% to 59.53% |
| Positive Predictive Value |  | 48.00% (*) | 36.31% to 59.85% |
| Negative Predictive Value |  | 50.00 % (*) | 11.81% to 88.19% |

S1Ka: Ong et al. 2010; Sleep Clinic Population - Severe OSA or AHI ≥30

| **Statistic** | **Formula** | **Value** | **95% CI** |
| --- | --- | --- | --- |
| Sensitivity |  | 95.58% | 89.98% to 98.55% |
| Specificity |  | 33.83 % | 27.32% to 40.82% |
| Positive Likelihood Ratio |  | 1.44 | 1.30 to 1.61 |
| Negative Likelihood Ratio |  | 0.13 | 0.05 to 0.31 |
| Disease prevalence |  | 35.99% (*) | 30.67% to 41.57% |
| Positive Predictive Value |  | 44.81% (*) | 38.43% to 51.33% |
| Negative Predictive Value |  | 93.15 % (*) | 84.74% to 97.74% |

S1La: Farney et al. 2011; Sleep Clinic Population - Severe OSA or AHI ≥30

| **Statistic** | **Formula** | **Value** | **95% CI** |
| --- | --- | --- | --- |
| Sensitivity |  | 95.69% | 93.70% to 97.19% |
| Specificity |  | 23.34 % | 20.53% to 26.34% |
| Positive Likelihood Ratio |  | 1.25 | 1.20 to 1.30 |
| Negative Likelihood Ratio |  | 0.18 | 0.12 to 0.28 |
| Disease prevalence |  | 40.73% (*) | 38.16% to 43.33% |
| Positive Predictive Value |  | 46.17% (*) | 43.33% to 49.04% |
| Negative Predictive Value |  | 88.74 % (*) | 83.83% to 92.58% |

S1Ma: El-Sayed et al. 2012; Sleep Clinic Population - Severe OSA or AHI ≥30

| **Statistic** | **Formula** | **Value** | **95% CI** |
| --- | --- | --- | --- |
| Sensitivity |  | 98.65% | 95.20% to 99.84% |
| Specificity |  | 4.88 % | 1.34% to 12.02% |
| Positive Likelihood Ratio |  | 1.04 | 0.98 to 1.09 |
| Negative Likelihood Ratio |  | 0.28 | 0.05 to 1.48 |
| Disease prevalence |  | 64.35% (*) | 57.79% to 70.53% |
| Positive Predictive Value |  | 65.18% (*) | 58.55% to 71.40% |
| Negative Predictive Value |  | 66.67 % (*) | 22.28% to 95.67% |

S1Na: Yu et al. 2012; Sleep Clinic Population - Severe OSA or AHI ≥30

| **Statistic** | **Formula** | **Value** | **95% CI** |
| --- | --- | --- | --- |
| Sensitivity |  | 100.00% | 92.29% to 100.00% |
| Specificity |  | 36.76 % | 25.39% to 49.33% |
| Positive Likelihood Ratio |  | 1.58 | 1.32 to 1.90 |
| Negative Likelihood Ratio |  | 0.00 |  |
| Disease prevalence |  | 40.35% (*) | 31.27% to 49.95% |
| Positive Predictive Value |  | 51.69% (*) | 40.84% to 62.41% |
| Negative Predictive Value |  | 100.00 % (*) | 86.28% to 100.00% |

S1Oa: Boynton et al. 2013; Sleep Clinic Population - Severe OSA or AHI ≥30

| **Statistic** | **Formula** | **Value** | **95% CI** |
| --- | --- | --- | --- |
| Sensitivity |  | 96.77% | 88.83% to 99.61% |
| Specificity |  | 33.12 % | 25.82% to 41.07% |
| Positive Likelihood Ratio |  | 1.45 | 1.28 to 1.63 |
| Negative Likelihood Ratio |  | 0.10 | 0.02 to 0.39 |
| Disease prevalence |  | 28.31% (*) | 22.45% to 34.77% |
| Positive Predictive Value |  | 36.36% (*) | 29.03% to 44.20% |
| Negative Predictive Value |  | 96.30 % (*) | 87.25% to 99.55% |

S1Pa: Pereira et al. 2013; Sleep Clinic Population - Severe OSA or AHI ≥30

| **Statistic** | **Formula** | **Value** | **95% CI** |
| --- | --- | --- | --- |
| Sensitivity |  | 96.43% | 87.69% to 99.56% |
| Specificity |  | 20.83 % | 12.16% to 32.02% |
| Positive Likelihood Ratio |  | 1.22 | 1.07 to 1.39 |
| Negative Likelihood Ratio |  | 0.17 | 0.04 to 0.72 |
| Disease prevalence |  | 43.75% (*) | 35.00% to 52.79% |
| Positive Predictive Value |  | 48.65% (*) | 39.05% to 58.32% |
| Negative Predictive Value |  | 88.24 % (*) | 63.56% to 98.54% |

S1Qa: Ha et al. 2014; Sleep Clinic Population - Severe OSA or AHI ≥30

| **Statistic** | **Formula** | **Value** | **95% CI** |
| --- | --- | --- | --- |
| Sensitivity |  | 86.27% | 73.74% to 94.30% |
| Specificity |  | 34.09 % | 24.32% to 44.97% |
| Positive Likelihood Ratio |  | 1.31 | 1.09 to 1.58 |
| Negative Likelihood Ratio |  | 0.40 | 0.19 to 0.85 |
| Disease prevalence |  | 36.69% (*) | 28.68% to 45.28% |
| Positive Predictive Value |  | 43.14% (*) | 33.37% to 53.32% |
| Negative Predictive Value |  | 81.08 % (*) | 64.84% to 92.04% |

S1Ra: Luo et al. 2014; Sleep Clinic Population - Severe OSA or AHI ≥30

| **Statistic** | **Formula** | **Value** | **95% CI** |
| --- | --- | --- | --- |
| Sensitivity |  | 97.66% | 93.30% to 99.51% |
| Specificity |  | 17.86 % | 10.35% to 27.74% |
| Positive Likelihood Ratio |  | 1.19 | 1.07 to 1.32 |
| Negative Likelihood Ratio |  | 0.13 | 0.04 to 0.44 |
| Disease prevalence |  | 60.38% (*) | 53.45% to 67.01% |
| Positive Predictive Value |  | 64.43% (*) | 57.26% to 71.16% |
| Negative Predictive Value |  | 83.33 % (*) | 58.58% to 96.42% |

S1Sa: Reis et al. 2015; Sleep Clinic Population - Severe OSA or AHI ≥30

| **Statistic** | **Formula** | **Value** | **95% CI** |
| --- | --- | --- | --- |
| Sensitivity |  | 98.39% | 91.34% to 99.96% |
| Specificity |  | 22.08 % | 15.80% to 29.46% |
| Positive Likelihood Ratio |  | 1.26 | 1.15 to 1.38 |
| Negative Likelihood Ratio |  | 0.07 | 0.01 to 0.52 |
| Disease prevalence |  | 28.70% (*) | 22.77% to 35.23% |
| Positive Predictive Value |  | 33.70% (*) | 26.86% to 41.09% |
| Negative Predictive Value |  | 97.14 % (*) | 85.08% to 99.93% |

S1Ta: Chung et al. 2008; Surgical Population - Severe OSA or AHI ≥30

| **Statistic** | **Formula** | **Value** | **95% CI** |
| --- | --- | --- | --- |
| Sensitivity |  | 100.00% | 90.97% to 100.00% |
| Specificity |  | 36.96 % | 28.90% to 45.58% |
| Positive Likelihood Ratio |  | 1.59 | 1.40 to 1.80 |
| Negative Likelihood Ratio |  | 0.00 |  |
| Disease prevalence |  | 22.03% (*) | 16.16% to 28.87% |
| Positive Predictive Value |  | 30.95% (*) | 23.02% to 39.80% |
| Negative Predictive Value |  | 100.00 % (*) | 93.02% to 100.00% |

S1Ua: Chung et al. 2012; Surgical Population - Severe OSA or AHI ≥30

| **Statistic** | **Formula** | **Value** | **95% CI** |
| --- | --- | --- | --- |
| Sensitivity |  | 94.78% | 89.53% to 97.87% |
| Specificity |  | 27.61 % | 24.11% to 31.34% |
| Positive Likelihood Ratio |  | 1.31 | 1.23 to 1.39 |
| Negative Likelihood Ratio |  | 0.19 | 0.09 to 0.39 |
| Disease prevalence |  | 17.96% (*) | 15.27% to 20.91% |
| Positive Predictive Value |  | 22.28% (*) | 18.93% to 25.92% |
| Negative Predictive Value |  | 96.02 % (*) | 91.98% to 98.39% |

S1Va: Firat et al. 2012; Other Miscellaneous study - Moderate to Severe OSA or AHI ≥15

| **Statistic** | **Formula** | **Value** | **95% CI** |
| --- | --- | --- | --- |
| Sensitivity |  | 86.96% | 73.74% to 95.06% |
| Specificity |  | 48.72 % | 32.42% to 65.22% |
| Positive Likelihood Ratio |  | 1.70 | 1.22 to 2.35 |
| Negative Likelihood Ratio |  | 0.27 | 0.12 to 0.60 |
| Disease prevalence |  | 54.12% (*) | 42.96% to 64.98% |
| Positive Predictive Value |  | 66.67% (*) | 53.31% to 78.31% |
| Negative Predictive Value |  | 76.00 % (*) | 54.87% to 90.64% |

S1Wa: Nicholl et al. 2013; Other Miscellaneous study - Moderate to Severe OSA or AHI ≥15

| **Statistic** | **Formula** | **Value** | **95% CI** |
| --- | --- | --- | --- |
| Sensitivity |  | 93.15% | 84.74% to 97.74% |
| Specificity |  | 30.30 % | 21.47% to 40.35% |
| Positive Likelihood Ratio |  | 1.34 | 1.16 to 1.54 |
| Negative Likelihood Ratio |  | 0.23 | 0.09 to 0.55 |
| Disease prevalence |  | 42.44% (*) | 34.95% to 50.20% |
| Positive Predictive Value |  | 49.64% (*) | 40.99% to 58.30% |
| Negative Predictive Value |  | 85.71 % (*) | 69.74% to 95.19% |

S1Xa: Silva et al. 2011; General Population - Moderate to Severe OSA or AHI ≥15

| **Statistic** | **Formula** | **Value** | **95% CI** |
| --- | --- | --- | --- |
| Sensitivity |  | 88.56% | 85.74% to 90.99% |
| Specificity |  | 29.93 % | 28.54% to 31.34% |
| Positive Likelihood Ratio |  | 1.26 | 1.22 to 1.31 |
| Negative Likelihood Ratio |  | 0.38 | 0.30 to 0.48 |
| Disease prevalence |  | 12.64% (*) | 11.71% to 13.62% |
| Positive Predictive Value |  | 15.46% (*) | 14.27% to 16.71% |
| Negative Predictive Value |  | 94.76 % (*) | 93.41% to 95.90% |

S1Ya: Nicholl et al. 2013; Other Miscellaneous study - Severe OSA or AHI ≥30

| **Statistic** | **Formula** | **Value** | **95% CI** |
| --- | --- | --- | --- |
| Sensitivity |  | 98.00% | 89.35% to 99.95% |
| Specificity |  | 27.05 % | 19.41% to 35.84% |
| Positive Likelihood Ratio |  | 1.34 | 1.20 to 1.51 |
| Negative Likelihood Ratio |  | 0.07 | 0.01 to 0.53 |
| Disease prevalence |  | 29.07% (*) | 22.41% to 36.47% |
| Positive Predictive Value |  | 35.51% (*) | 27.55% to 44.10% |
| Negative Predictive Value |  | 97.06 % (*) | 84.67% to 99.93% |

S1Za: Silva et al. 2011; General Population - Severe OSA or AHI ≥30

| **Statistic** | **Formula** | **Value** | **95% CI** |
| --- | --- | --- | --- |
| Sensitivity |  | 92.75% | 89.49% to 95.26% |
| Specificity |  | 29.18 % | 27.84% to 30.54% |
| Positive Likelihood Ratio |  | 1.31 | 1.26 to 1.36 |
| Negative Likelihood Ratio |  | 0.25 | 0.17 to 0.36 |
| Disease prevalence |  | 7.23% (*) | 6.51% to 8.00% |
| Positive Predictive Value |  | 9.26% (*) | 8.32% to 10.28% |
| Negative Predictive Value |  | 98.10 % (*) | 97.21% to 98.77% |
